# *Helicobacter pylori*-controlled c-Abl localization promotes cell migration and limits apoptosis

**DOI:** 10.1186/s12964-019-0323-9

**Published:** 2019-01-31

**Authors:** Gernot Posselt, Maria Wiesauer, Bianca E. Chichirau, Daniela Engler, Linda M. Krisch, Gabriele Gadermaier, Peter Briza, Sabine Schneider, Francesco Boccellato, Thomas F. Meyer, Cornelia Hauser-Kronberger, Daniel Neureiter, Anne Müller, Silja Wessler

**Affiliations:** 1 Department of Biosciences, Division of Microbiology, University of Salzburg, Paris-Lodron University of Salzburg, Billroth Str. 11, A-5020 Salzburg, Austria; 2 Institute of Molecular Cancer Research, University of Zurich, Winterthurerstrasse 190, CH-8057 Zürich, Switzerland; 3 Department of Biosciences, Division of Allergy and Immunology, University of Salzburg, Paris-Lodron University of Salzburg, Hellbrunner Str. 34, A-5020 Salzburg, Austria; 4 Paul-Ehrlich-Institute, Paul-Ehrlich-Str. 51-59, D-63225 Langen, Germany; 5 Max Planck Institute for Infection Biology, Charitéplatz 1, D-10117 Berlin, Germany; 6 Department of Pathology, Paracelsus Medical University Salzburg, Müllner Hauptstraße 48, A-5020 Salzburg, Austria; 7 Cancer Cluster Salzburg, University of Salzburg, A-5020 Salzburg, Austria

**Keywords:** C-Abl, Apoptosis, Cancer, Gastritis, *Helicobacter pylori*, Motility, βHBP, PKC

## Abstract

**Background:**

Deregulated c-Abl activity has been intensively studied in a variety of solid tumors and leukemia. The class-I carcinogen *Helicobacter pylori* (*Hp*) activates the non-receptor tyrosine kinase c-Abl to phosphorylate the oncoprotein cytotoxin-associated gene A (CagA). The role of c-Abl in CagA-dependent pathways is well established; however, the knowledge of CagA-independent c-Abl processes is scarce.

**Methods:**

c-Abl phosphorylation and localization were analyzed by immunostaining and immunofluorescence. Interaction partners were identified by tandem-affinity purification. Cell elongation and migration were analyzed in transwell-filter experiments. Apoptosis and cell survival were examined by FACS analyses and MTT assays. In mice experiments and human biopsies, the involvement of c-Abl in *Hp* pathogenesis was investigated.

**Results:**

Here, we investigated the activity and subcellular localization of c-Abl in vitro and in vivo and unraveled the contribution of c-Abl in CagA-dependent and -independent pathways to gastric *Hp* pathogenesis. We report a novel mechanism and identified strong c-Abl threonine 735 phosphorylation (pAbl^T735^) mediated by the type-IV secretion system (T4SS) effector D-glycero-β-D-manno-heptose-1,7-bisphosphate (βHBP) and protein kinase C (PKC) as a new c-Abl kinase. pAbl^T735^ interacted with 14–3-3 proteins, which caused cytoplasmic retention of c-Abl, where it potentiated *Hp*-mediated cell elongation and migration. Further, the nuclear exclusion of pAbl^T735^ attenuated caspase-8 and caspase-9-dependent apoptosis. Importantly, in human patients suffering from *Hp*-mediated gastritis c-Abl expression and pAbl^T735^ phosphorylation were drastically enhanced as compared to type C gastritis patients or healthy individuals. Pharmacological inhibition using the selective c-Abl kinase inhibitor Gleevec confirmed that c-Abl plays an important role in *Hp* pathogenesis in a murine in vivo model.

****Conclusions**:**

In this study, we identified a novel regulatory mechanism in *Hp*-infected gastric epithelial cells by which *Hp* determines the subcellular localization of activated c-Abl to control *Hp*-mediated EMT-like processes while decreasing cell death.

**Electronic supplementary material:**

The online version of this article (10.1186/s12964-019-0323-9) contains supplementary material, which is available to authorized users.

## Background

*Helicobacter pylori* (*Hp*) is a human class-I carcinogen that exclusively colonizes the gastric epithelium of approximately 50% of the world’s population. Successful *Hp* colonization requires sophisticated strategies to survive the hostile gastric environment and to prevent clearance by the immune system. Persistent infections with *Hp* are considered as the main factor responsible for chronic gastritis, ulceration, lymphoma of the MALT system and gastric cancer [1, 2]. While MALT lymphoma can be treated by antibiotics as the first line therapy, the prognosis of gastric cancer is still poor and represents one of the leading causes for cancer-related deaths worldwide. Surgery is the only curative treatment, since chemo-, radiation-, or targeted therapies are not efficient in advanced stages of gastric cancer and fail to prevent epithelial-mesenchymal transition (EMT)-driven tumor spreading [3]. Gastric cancer can be distinguished in cardia (gastro-esophageal junction) and non-cardia adenocarcinomas caused by altered cell proliferation, survival, apoptosis and (epigenetic) modifications of tumor suppressor genes (*cdh1*, *tp53*, *kras*, etc.) [3, 4]. Hence, *Hp*-mediated tumorigenesis and gastric cancer progression involve a complex network of signaling cascades which allows persistent colonization and causes the induction of inflammatory and carcinogenic responses.

The genome of highly virulent *Hp* strains harbors a cag pathogenicity island (*cag*PAI), which encodes a specialized type-4 secretion system (T4SS). Via the T4SS pilus, *Hp* translocates the effector protein CagA into the cytoplasm of gastric epithelial cells [5, 6]. CagA is initially tyrosine phosphorylated (pCagA) in its Glu-Pro-Ile-Tyr-Ala (EPIYA) motifs by members of the Src kinase family [7, 8] followed by phosphorylation through c-Abl to maintain pCagA in later phases of *Hp* infections [9, 10]. In fact, pCagA is considered as an important driver of oncogenic processes. Transgenic mice systemically expressing CagA suffer from gastric epithelial hyperplasia, gastric polyps, hematological malignancies and adenocarcinomas. This report provides a direct and causative link between pCagA and the development of *Hp-*associated neoplasms [11].

The pathogenic function of CagA has been demonstrated in vivo in animal models [12, 13] and in cultured gastric epithelial cells in vitro [14–16]. *Hp*-infected AGS cells display a strongly elongated cell morphology resembling the cell scattering phenotype in response to hepatocyte growth factor (HGF) [17, 18]. *Hp*-mediated cell elongation is strictly dependent on Src- and c-Abl-mediated CagA phosphorylation [9, 10] and is associated with the CagA-independent loss of intercellular adhesion and enhanced cell migration. These processes are implicated in the development of an EMT-like phenotype, which represents a critical step during metastasis [19].

The non-receptor tyrosine kinase c-Abl exhibits manifold cellular functions and its structure and regulation have been well studied [20, 21]. It contains protein-protein interaction domains, DNA- and actin-binding motifs, nuclear localization signals (NLS) and nuclear export signals (NES). The kinase activity can be activated by numerous pathways including platelet-derived growth factor receptor (PDGFR), epidermal growth factor receptor (EGFR) or through substrate interaction [22] and is accompanied by phosphorylation at tyrosine 245 (pAbl^Y245^) and tyrosine 412 (pAbl^Y412^) [21, 23]. The consequences of kinase activation range from cytoskeleton rearrangements, cell motility, and proliferation to DNA damage response and apoptotic pathways [24, 25]. These opposing effects are mainly regulated via the subcellular localization of the kinase. NLS and NES sequences regulate shuttling of c-Abl between the cytoplasm and the nucleus. In the cytoplasm, c-Abl is involved in the regulation of actin dynamics and proliferation. Accordingly, many of the identified kinase substrates (e.g. Crk proteins, cortactin, Wave, etc.) are closely associated with cell morphology and migration [22, 26]. In contrast, nuclear c-Abl contributes to the DNA damage response [24] and apoptosis [27, 28]. Therefore, a balanced nucleo-cytoplasmic transport of c-Abl is a tightly regulated process in normal cells. c-Abl expression, activity and localization are frequently deregulated in human leukemia, but also in solid tumors, and is implicated in neoplastic transformation and cancer progression [29, 30]. It has been shown that cytoplasmic localization is mainly regulated by interaction with members of the 14–3-3 protein family, which preferentially bind to phosphorylated threonine 735 (pAbl^T735^) and thereby mask the NLS motifs [31, 32]. The drastic consequences of cytoplasmic Abl kinase activity are displayed by the oncogenic breakpoint cluster region (BCR)-Abl fusion protein. A vast majority of chronic myeloid leukemia (CML) cases are caused by the Philadelphia translocation, which results in a constitutively active BCR-Abl representing the paradigm of therapeutic intervention using specific kinase inhibitors [20, 33].

Gastric cancer cells can leave the primary tumor, invade the surrounding extracellular matrix (ECM), and metastasize to distal sites; however, it is not fully understood how these invasive cells survive in a foreign environment. These processes likely involve the inactivation of apoptotic mechanisms and uncontrolled proliferation. In our previous work, we identified c-Abl as a crucial molecule for CagA functions in *Hp*-infected gastric epithelial cells [9, 10]. Besides its influence on CagA, the cellular consequences of activated c-Abl are largely unknown. Hence, we analyzed how *Hp* controls c-Abl subcellular localization and influences cell fate in vitro and in vivo.

## Methods

### Cell and bacterial culture

The gastric epithelial cancer cell lines AGS (ECACC, no. 89090402) and MKN-28 (MPI for Infection Biology in Berlin, JCRB, no. 0253) were cultured in RPMI-1640 (Sigma Aldrich, Vienna, Austria) containing 10% FCS (Sigma Aldrich, Austria) and 2 mM L-glutamine (Biowest, France) at 37 °C in a humidified 5% CO_2_ atmosphere. MCF-7 cells (ATCC, no. HTB-22) were cultured in DMEM medium (Sigma Aldrich, Austria) containing 10% FCS and 2 mM L-glutamine at 37 °C in a humidified 10% CO_2_ atmosphere. *Hp* P12 wildtype was cultured on horse serum agar plates for 24 to 48 h at 37 °C under microaerophilic conditions using the CampyGen system (Oxoid, Austria). *Hp* P12 wt, ΔPAI, ΔCagA, ΔVacA, ΔRfaE, ΔCagL and ΔCagL/CagL isogenic mutant strains have been described previously [34–37]. Additional Western (P1, Hp26695, and B8) and East Asian isolates (42GX, 48GX) of *Hp* has been reported elsewhere [38–41]. *Hp* was harvested in PBS, pH 7.4 (Sigma Aldrich, Austria) and added to host cells at a multiplicity of infection (MOI) as indicated. Cells were routinely serum starved for one hour before infection. Where indicated, cells were stimulated with 100 nM phorbol-12-myristat-13-acetat (PMA, Sigma Aldrich, Austria), 10 μM H_2_O_2_/100 μM sodium vanadate, 10 μM of the 14–3-3 inhibitor BV02 (Sigma-Aldrich, Austria), or pretreated with 10 μM STI-571 (LC Laboratories, MA, USA) to block c-Abl. To inhibit protein kinase A (PKA) activity, 10 μM PKI (Sigma Aldrich, Austria) was used. PKC inhibitors Gö6983 and BIM have been described elsewhere [42] and were obtained from Sigma Aldrich (Austria).

### DNA constructs and transfection

The plasmids pSGT-Abl^wt^, pSGT-Abl^KD^ (K290R) and pSGT-Abl^PP^ (P242E, P249E) have been described previously [43]. The constructs pSGT-Abl^TA^, pSGT-Abl^Y245F^, pSGT-Abl^Y412F^, pNTAP-Abl^wt^, and pNTAP-Abl^TA^ have been generated by site directed mutagenesis (Quikchange Lightning, Agilent Technologies, Germany). All constructs were verified by sequencing.

### Transient transfection, siRNA and generation of stable cell lines

Cells were transfected with 5 μg plasmid using polyethylenimine (Polysciences Europe, Germany). For the generation of stable cell lines, AGS cells were transfected with linearized pNTAP-Abl^wt^ and pNTAP-Abl^TA^ plasmids and selected using G418 (Sigma-Aldrich, Austria). Generation of stable shAbl knock-down cells and the corresponding negative control (sh control) has been described previously [10]. For siRNA knock-down experiments, siTTK, siPKC (further information in the Additional file 1) and control siRNA oligonucleotides (Santa Cruz Biotechnology, Germany) were co-transfected with pSGT-Abl^wt^. After 3 days, cells were infected with *Hp* and analyzed by Western blotting.

### SDS PAGE and Western blot

Cells were lysed in 20 mM Tris pH 7.5, 1 mM EDTA, 100 mM NaCl, 1% Triton X-100, 0.1% SDS, 1 x complete protease inhibitor tablets (Roche Diagnostics, Germany), 1 mM sodium molybdate, 20 mM NaF, 10 mM sodium pyrophosphate, 20 mM β-glycerophosphate, 1 mM sodium vanadate. Equal protein amounts were separated by SDS PAGE and transferred onto nitrocellulose. Following antibodies were used: anti-c-Abl (AB3, Merck Biosciences, Germany), anti-pAbl^T735^, anti-pCrkII^Y221^ (both New England Biolabs, Germany), anti-pAbl^Y245^, anti-β-actin (both Sigma Aldrich, Germany), anti-pAbl^Y412^, anti-GAPDH (both Abcam, UK), anti-CagA [44], anti-GST (Biomol Germany), anti-14-3-3 H8, anti-phospho-tyrosine (pY99), anti-TTK, and anti-PKC (all Santa Cruz Biotechnology, Germany). Membranes were imaged using the Molecular Imager ChemiDoc XRS system (BioRad, Germany). Where indicated, signals of protein bands were quantified using the ImageLab software (BioRad, Germany).

### Immunoprecipitation, in vitro kinase reaction and TAP pull down experiments

c-Abl was precipitated from 500 μg whole cell lysates using 3 μg anti-c-Abl (AB3, Merck Biosciences, Germany). The in vitro kinase reaction was performed in 20 mM HEPES pH 7.4, 10 mM MgCl_2_, 10 mM MnCl_2_, 250 μM ATP using 250 ng purified GST-Crk aa 120–225 [26] for 30 min at 30 °C. PKC-mediated c-Abl phosphorylation was performed using 10 ng/μl recombinant PKCαβγ (Merck Millipore, Germany), 100 ng/μl recombinant c-Abl (Merck Millipore, Germany) and 250 μM ATP for 10 min at 30 °C in an assay dilution buffer II reaction buffer (Merck Millipore, Germany). To activate PKC activity, a PKC lipid activator (Merck Millipore, Germany) has been added to the reaction as recommend by manufacturer’s instructions. TAP pull-downs were performed using the Interplay Mammalian TAP System (Agilent Technologies, Austria) according to the manufacturer’s manual.

### Immunofluorescence

Cells were grown on coverslips, transfected with pSGT-Abl^wt^ or pSGT-Abl^TA^ and infected for the indicated periods of time. Cells were washed twice with PBS, fixed in 4% paraformaldehyde, permeabilized with 0.2% Triton X-100, followed by blocking in 1% bovine serum albumin (BSA). Cells were stained using 0.5 μg/ml anti-c-Abl (AB3, Merck Biosciences, Germany). Cells were counterstained with phalloidin-Alexa-Fluor546 (ThermoFisher Scientific, Austria) and DAPI (Sigma Aldrich, Austria). Imaging was performed using an AxioObserver Z1 (Zeiss, Austria). Cell elongation was determined by measuring the largest cell diameter using ZEN2 (Zeiss) and Fiji software. c-Abl-positive cells were measured in 4–6 random frames per experiment from four independent infection experiments. Nuclear and cytoplasmic localization of c-Abl was quantitated by measuring the integrated intensities of the nuclear and cytoplasmic areas stained by DAPI and phalloidin in 4–6 random frames per experiment from four independent infection experiments. The cytoplasmic c-Abl was calculated as total cell c-Abl intensity (set as 100%) minus nuclear c-Abl intensity.

### Quantification of cell migration

AGS cells stably expressing TAP-Abl^wt^ or TAP-Abl^TA^ were seeded in 8 μm transwell filter inserts (BD Biosciences, Austria). Next day, the medium was replaced by RPMI supplemented with 1% FCS. After 24 h, cells were infected with *Hp* at a MOI 50 for 8 h. Non-migrating cells were removed and migrating cells were stained by Giemsa and counted.

### Apoptosis and MTT assay

Apoptosis was measured using PE annexin V Apoptosis Detection Kit I (BD Biosciences, Austria). Caspase-8 and caspase-9 activation was determined using the Milliplex human early apoptosis kit (Millipore, Germany). For MTT assays, cells were incubated with 0.5 mg/ml 3-(4,5-Dimethylthiazol-2-yl)-2,5-diphenyltetrazolium bromide (Sigma-Aldrich, Austria) for 1 h at 37 °C in the dark. Cells were lysed using isopropylalcohol containing 0.1% NP-40 and 0.04 N HCl. Absorbance was read at 565 nm in a Tecan M200 plate reader.

### Mouse colonization experiments

C57BL/6 mice were infected with 10^7^ *Hp* strain PMSS1 for two months. Mice were either left untreated or were treated with 75 mg/kg per day STI-571 in the drinking water. Colony-forming units (*cfu*) were determined by plating and colony counting. Paraffin sections were stained with hematoxylin and eosin (H&E) for grading of histopathological changes. Details can be found in the Additional file 1.

### Immunohistochemistry

Immunohistochemical staining of human gastric biopsies for c-Abl and pAbl^T735^ was performed on routinely FFPE tissue, using a standardized automated platform (AutostainerPlus, Dako, DN) in combination with Envision polymer detection system (Agilent Technologies, Austria). Details can be found in the Additional file 1.

### Statistics

Statistical evaluations for cell culture experiments were calculated using Student’s *t*-test with GraphPad Prism 5. For the animal experiments statistics was calculated using Wilcoxon-Mann-Whitney test with GraphPad Prism 5. Statistics for the human gastric biopsy specimen was calculated using Bonferroni corrected least significant difference test using SPSS software.

## Results

### *Hp* regulates c-Abl tyrosine and threonine phosphorylation via different signaling pathways

**Fig. 1:**
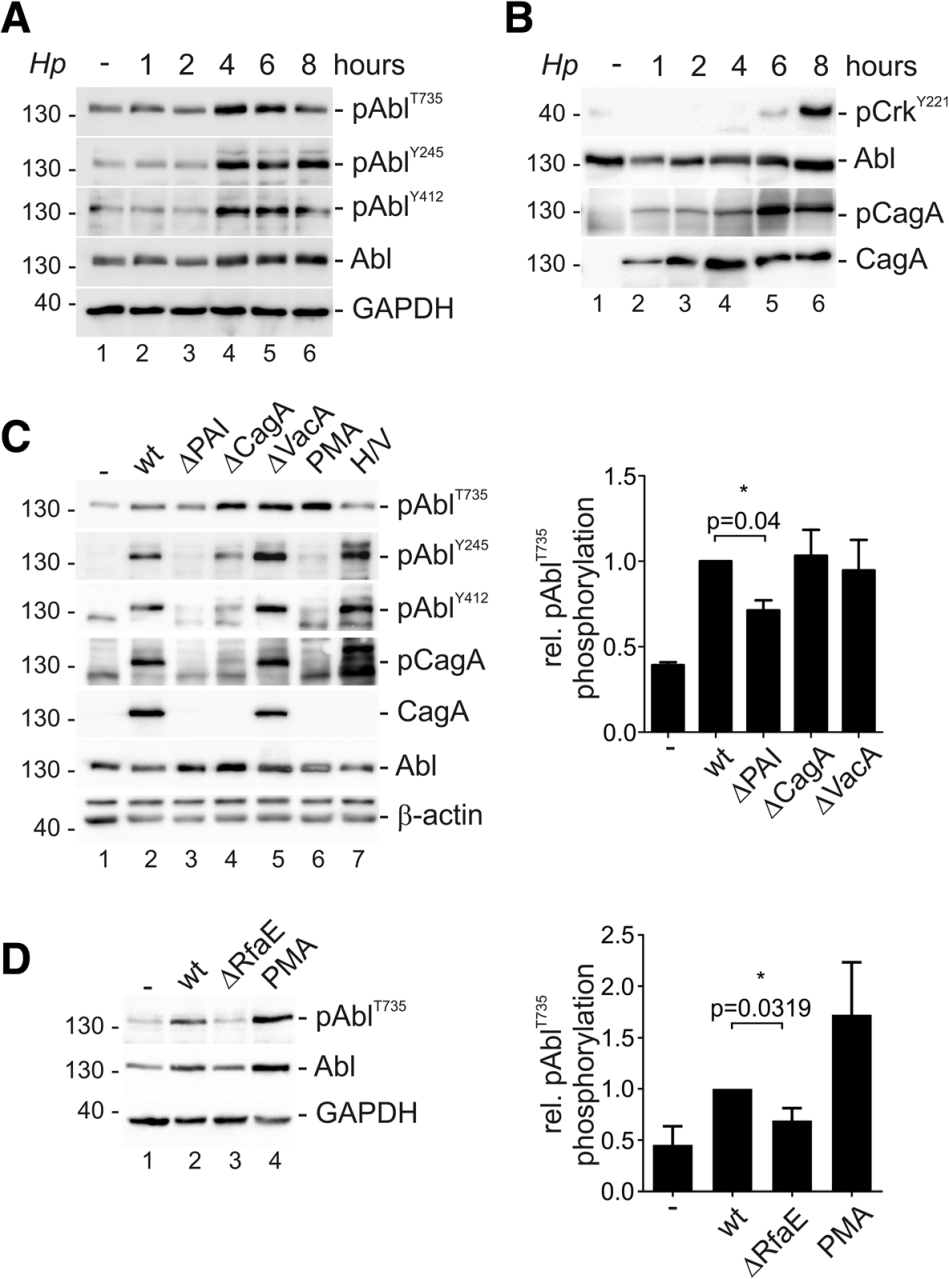
*Hp-*induced threonine phosphorylation and kinase activity of c-Abl. pSGT-Abl^wt^-transfected AGS cell were infected with *Hp* as indicated. **(a)** Phosphorylation of pAbl^T735^, pAbl^Y245^ and pAbl^Y412^ was analyzed by using phospho-specific antibodies. Abl and GAPDH were shown as loading controls. **(b)** To analyze the kinase activity, c-Abl was immunoprecipitated and incubated with recombinant GST-Crk as a substrate. Crk phosphorylation was demonstrated using an anti-phospho-Crk^Y221^ antibody. In whole cell lysates, pCagA, Abl and total CagA were detected as controls. **(c)** AGS cells were infected with *Hp* wt or ΔPAI, ΔCagA, or ΔVacA mutants to investigate pAbl^T735^, pAbl^Y245^, pAbl^Y412^ and Abl. Where indicated, cells were treated with PMA or H_2_O_2_/sodium vanadate (H/V). Translocated pCagA, CagA and β-actin were shown as controls (left panel). The relative amounts of pAbl^T735^, pAbl^Y245^ and pAbl^Y412^ signals were quantified by blot densitometry and normalized to the loading control (right panel). **(d)** Cells were infected with *Hp* wt, ΔRfaE or treated with PMA. pAbl^T735^, Abl and GAPDH were detected using specific antibodies (left panel). The relative amounts of pAbl^T735^, pAbl^Y245^ and pAbl^Y412^ signals were quantified by blot densitometry and normalized to the loading control. These results are presented as relative phosphorylation with the levels induced by *Hp* (wt) set to 1.0 (right panel)

Although c-Abl plays a well documented crucial role in *Hp* pathogenesis, the complex network of kinase regulation has not been investigated in detail. To analyze the regulatory phosphorylation sites, c-Abl was transiently transfected to facilitate the detection of phospho-c-Abl. For the first time, we could show pAbl^T735^ phosphorylation in *Hp*-infected cells, which was weak in non-infected AGS cells, but strongly induced after 4 and 6 h of *Hp* infection. Concomitantly, *Hp* induced the phosphorylation of pAbl^Y245^ and pAbl^Y412^, but also a slight increase of c-Abl protein amount was observed (Fig. 1a), which has been previously reported and was attributed to miRNA-203 silencing [45]. The amounts of c-Abl and pAbl^T735^ in *Hp*-infected cells were quantified and correlated with non-infected cells. A drastic increase in pAbl^T735^ phosphorylation was observed, which outbalanced the minor effects of c-Abl accumulation (Additional file 2: Figure S1A), underlining that *Hp* effectively induced pAbl^T735^ phosphorylation. This could also be detected in MKN28 (Additional file 2: Figure S1B) and MCF-7 cells (Additional file 2: Fig. S1C), which have been established as suitable *Hp* infection models [46]. MKN28 cells, which express higher levels of endogenous c-Abl [47], were analyzed by immunoprecipitation to detect endogenous pAbl^T735^ upon *Hp* infection (Additional file 2: Figure S1D). We further analyzed multiple Western and East Asian *Hp* isolates and observed a robust pAbl^T735^ phosphorylation (Additional file 2: Figure S1E). In line with the detected tyrosine phosphorylation pattern, c-Abl kinase activity was strongly activated at later time points after *Hp* infection as reflected by the phosphorylation of the c-Abl substrate GST-Crk in in vitro phosphorylation assays (Fig. 1b). A set of various isogenic *Hp* deletion mutants was analyzed, which *Hp* factors are involved in the regulation of c-Abl. CagA is encoded by the *cag* pathogenicity island (*cag*PAI) which also harbors the genes important for the structure and function of the T4SS including the T4SS adhesin CagL [5]. The vacuolating toxin VacA has been described as an inducer of vacuolization and apoptosis [48]. In comparison to *Hp* wildtype (wt), a *∆cag*PAI-deficient strain failed to mediate pAbl^Y245^ or pAbl^Y412^ phosphorylation. This is in contrast to pAbl^T735^, which was only partially affected by the deletion of the *cag*PAI (Fig. 1c). Therefore, we investigated whether the T4SS adhesin CagL triggers pAbl^T735^ phosphorylation. CagL expression in *Hp* wildtype and the complemented ∆CagL mutant was necessary for phosphorylation of pAbl^Y245^ or pAbl^Y412^, but not for pAbl^T735^ (Additional file 3: Figure S2A-B). In addition, CagA deficiency resulted in a slight decrease in c-Abl tyrosine phosphorylation, but exhibited no effect on the pAbl^T735^ phosphorylation. Finally, loss of VacA expression did not influence pAbl^T735^ phosphorylation, but increased pAbl^Y245^ or pAbl^Y412^ phosphorylation. Detection of CagA and translocated pCagA validated *Hp* mutants (Fig. 1c). Recently, βHBP was identified as a new T4SS effector [37, 49]. The *rfaE-*deficient *Hp* mutant exhibits a defect in the HBP biosynthesis and did not mediate pAbl^T735^ phosphorylation while pAbl^Y245^ was still induced (Fig. 1d and Additional file 3: Figure S2C). Additionally, cells were stimulated with PMA or H_2_O_2_/vanadate serving as positive controls for phosphorylation of pAbl^T735^, pAbl^Y245^ and pAbl^Y412^, respectively (Fig. 1c-d). These data suggest that the T4SS-dependent βHBP effector is implicated in the control of c-Abl threonine phosphorylation while tyrosine phosphorylation and activation of c-Abl is CagL/CagA-dependent.

To analyze whether phosphorylation of pAbl^T735^ is linked to pAbl^Y245^ or pAbl^Y412^, a panel of c-Abl mutants targeting kinase activity and phosphorylation sites was generated for a detailed characterization of their potential mutual regulation. We created phosphorylation-resistant mutants of threonine 735 (Abl^TA^), tyrosine 245 (Abl^Y245F^) and tyrosine 412 (Abl^Y412F^) and analyzed them together with constitutively active (Abl^PP^) and a kinase-dead version of c-Abl (Abl^KD^) in Western blot and densitometric analyses (Additional file 4: Figure S3A-D). Compared to Abl^wt^, neither Abl^Y245F^ nor Abl^Y412F^ or Abl^KD^ significantly affected pAbl^T735^ phosphorylation. As expected, Abl^TA^ expression completely abrogated pAbl^T735^ phosphorylation signals (Additional file 4: Figure S3A, right panel and S3B). Corresponding to the Abl^Y245F^ or Abl^Y412F^ mutants, treatment of AGS cells with STI-571 efficiently blocked pAbl^Y245^ phosphorylation, but did not change pAbl^T735^ phosphorylation (Additional file 4: Figure S3E). The analyses of pAbl^Y245^ or pAbl^Y412^ phosphorylation verified the functionality of the respective mutants. The lack tyrosine 245 phosphorylation of Abl^PP^ is due to the exchange of the prolines 242 and 249 to glutamates, which interferes with pAbl^Y245^ phosphorylation (Additional file 4: Figure S3A, left panel). Importantly, pAbl^Y245^ and pAbl^Y412^ were hyper-phosphorylated in cells transfected with the Abl^TA^ construct (Additional file 4: Figure S3A, left panel and S3C-D). Interestingly, pAbl^Y245^ was also abrogated in the Abl^Y412F^ mutant pointing to a hierarchical phosphorylation of these sites. In contrast, pAbl^Y412^ was unaffected by the Abl^Y245F^ mutant (Additional file 4: Figure S3A, left panel and Additional file 4: Figure S3C-D). Further, overexpression of Abl^wt^ induced an increase in CagA phosphorylation, while cells expressing Abl^TA^ exhibited an attenuated pCagA signal (Additional file 4: Figure S3A, right panel). Unsurprisingly, constitutive active Abl^PP^ strongly increased pCagA, whereas Abl^KD^, Abl^Y245F^, and Abl^Y412F^ clearly reduced pCagA signals (Additional file 4: Fig. S3A, right panel). The data imply that pAbl^T735^ and pAbl^Y245^ phosphorylations are induced by different *Hp* factors and upstream signal transduction pathways.

### PKC is a novel kinase for phosphorylation of pAbl^T735^ which causes cytoplasmic retention, increases cell migration and limits apoptosis

**Fig. 2:**
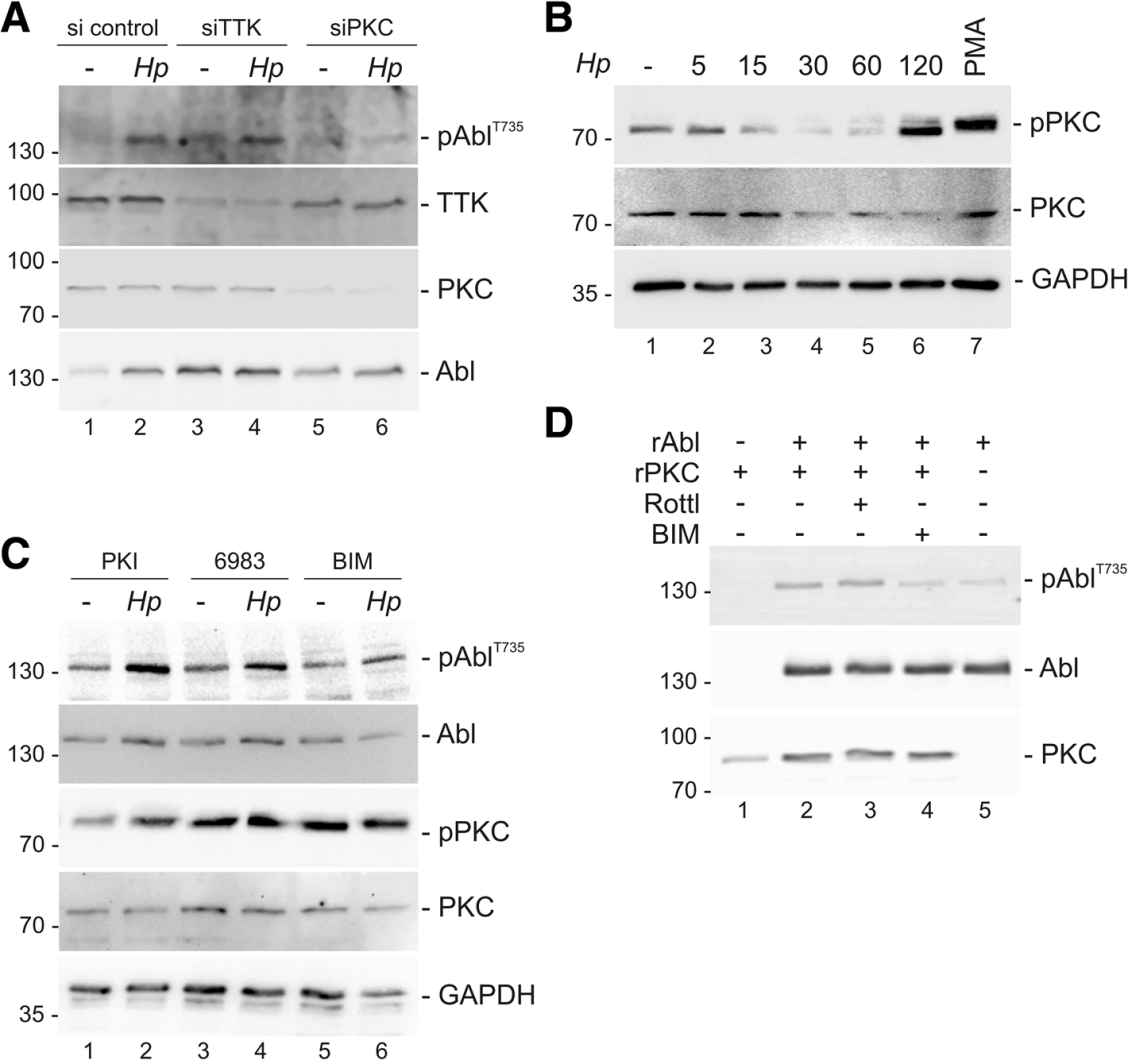
PKC induces pAbl^T735^ phosphorylation. **(a)** AGS cells were cotransfected with pSGT-Abl^wt^ and si control, siTTK or siPKC and were left untreated or infected with *Hp* for 6 h. Lysates were analyzed for pAbl^T735^, TTK, PKC and Abl. **(b)** AGS cells were left untreated, infected with *Hp* or stimulated with 100 nM PMA. Lysates were analyzed for pPKC, PKC and GAPDH. **(c)** pSGT-Abl^wt^-transfected AGS cells were treated with a PKA inhibitor (PKI) as a negative control or the PKC inhibitors Gö6983 and BIM. Where indicated, cells were infected with *Hp* and analyzed for pAbl^T735^, Abl, pPKC, PKC and GAPDH. **(d)** Recombinant c-Abl (rAbl) and PKCα/β/γ (rPKC) were co-incubated with rottlerin (Rottl) or BIM. pAbl^T735^, Abl and PKC proteins were detected

In previous studies TTK/Mps1 has been proposed to mediate phosphorylation of pAbl^T735^ [32]. In addition, online kinase prediction tools (NetPhos, http://www.cbs.dtu.dk/services/NetPhos/) yielded PKC as putative pAbl^T735^ kinase. Therefore, we tested the influence of both, TTK and PKC by knock-down of protein expression using specific siRNA. TTK-targeting siRNA resulted in efficient down-regulation of TTK expression; however, *Hp*-induced pAbl^T735^ was not affected as compared to control siRNA (Fig. 2a). In contrast, PKC knock-down strongly inhibited the phosphorylation of pAbl^T735^ (Fig. 2a). PKC activation has previously been shown to play a crucial role in *Hp*-mediated cell elongation and scattering [42]. In fact, *Hp* induced a robust PKC activation (Fig. 2b). To confirm that PKC plays a direct role in the upstream signaling of pAbl^T735^, PKC activity was blocked using Gö6983 and BIM. In contrast to the protein kinase A inhibitor (PKI) used as a negative control, Gö6983 slightly affected phosphorylation of pAbl^T735^, while bis(indolyl)maleimide (BIM) drastically reduced the phosphorylation of pAbl^T735^ (Fig. 2c). Even though the inhibitors Gö6983 and BIM enhanced the basal level of PKC phosphorylation, which has been observed previously [42], *Hp* did not further stimulate an increase in pPKC (Fig. 2c). In an in vitro kinase assay, recombinant PKCα/β/γ (rPKC) directly phosphorylated recombinant c-Abl (rAbl), which was again blocked by BIM, but not by the PKCδ-specific inhibitor rottlerin (Fig. 2d). Since PKCγ expression is restricted to neuronal cells [50], these data point to PKCα/β as *Hp*-regulated kinases that directly phosphorylate pAbl^T735^ in gastric epithelial cells.

**Fig. 3:**
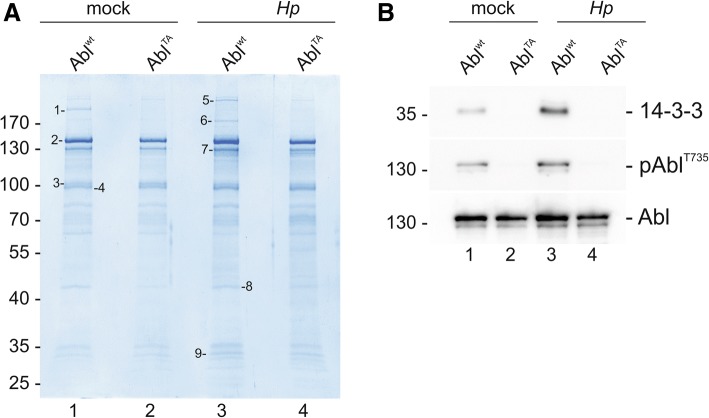
Identification of 14–3-3 as c-Abl interaction partner. TAP-Abl^wt^ or TAP-Abl^TA^-transfected AGS cells were left untreated (mock) or infected with *Hp* for 6 h. (**a)** After TAP experiment, proteins were detected by coomassie-stained SDS-PAGE. Protein bands 1–9 showing differential interaction with c-Abl were identified by mass spectrometry. **(b)** Aliquots of samples were examined by Western blot to verify *Hp*-regulated 14–3-3/pAbl^T735^ interaction. TAP-Abl indicates equal protein loading

To identify potential interaction partners of c-Abl in *Hp*-infected cells tandem-affinity purification (TAP) experiments were performed. Differential *Hp*-dependent binding patterns were observed in TAP-Abl^wt^- and TAP-Abl^TA^-expressing cells (Fig. 3a). Phosphorylation of TAP-Abl^wt^ and TAP-Abl^TA^ was verified by Western blotting (Additional file 5: Figure S4A). Candidate proteins were then analyzed by mass-spectrometry and members of the 14–3-3 family were identified (Table 1). In line with pAbl^T735^ signals, a weak interaction of c-Abl^wt^ with 14–3-3 was observed in non-infected cells and binding was drastically increased upon infection with *Hp*. This interaction was completely abolished in cells expressing c-Abl^TA^ (Fig. 3b).

**Table 1:** Identified c-Abl interaction partners in *Hp*-infected cells

Sample #	Accession	Description	Score	# Peptides
1	P35579	Myosin-9	148,23	50
2	P00519	Tyrosine-protein kinase ABL1	842,98	47
3	P00519	Tyrosine-protein kinase ABL1	300,10	30
4	P00519	Tyrosine-protein kinase ABL1	219,43	34
5	Q01082	Spectrin beta chain, non-erythrocytic 1	544,78	95
6	P46940	Ras GTPase-activating-like protein IQGAP1	315,66	62
7	P00519	Tyrosine-protein kinase ABL1	624,71	38
8	B4DVQ0	cDNA FLJ58286, highly similar to Actin, cytoplasmic 2	90,94	8
9	P61981	14–3-3 protein gamma	18,73	5
	Q04917	14–3-3 protein eta	24,49	9
	P31946	14–3-3 protein beta/alpha	21,49	7

**Fig. 4:**
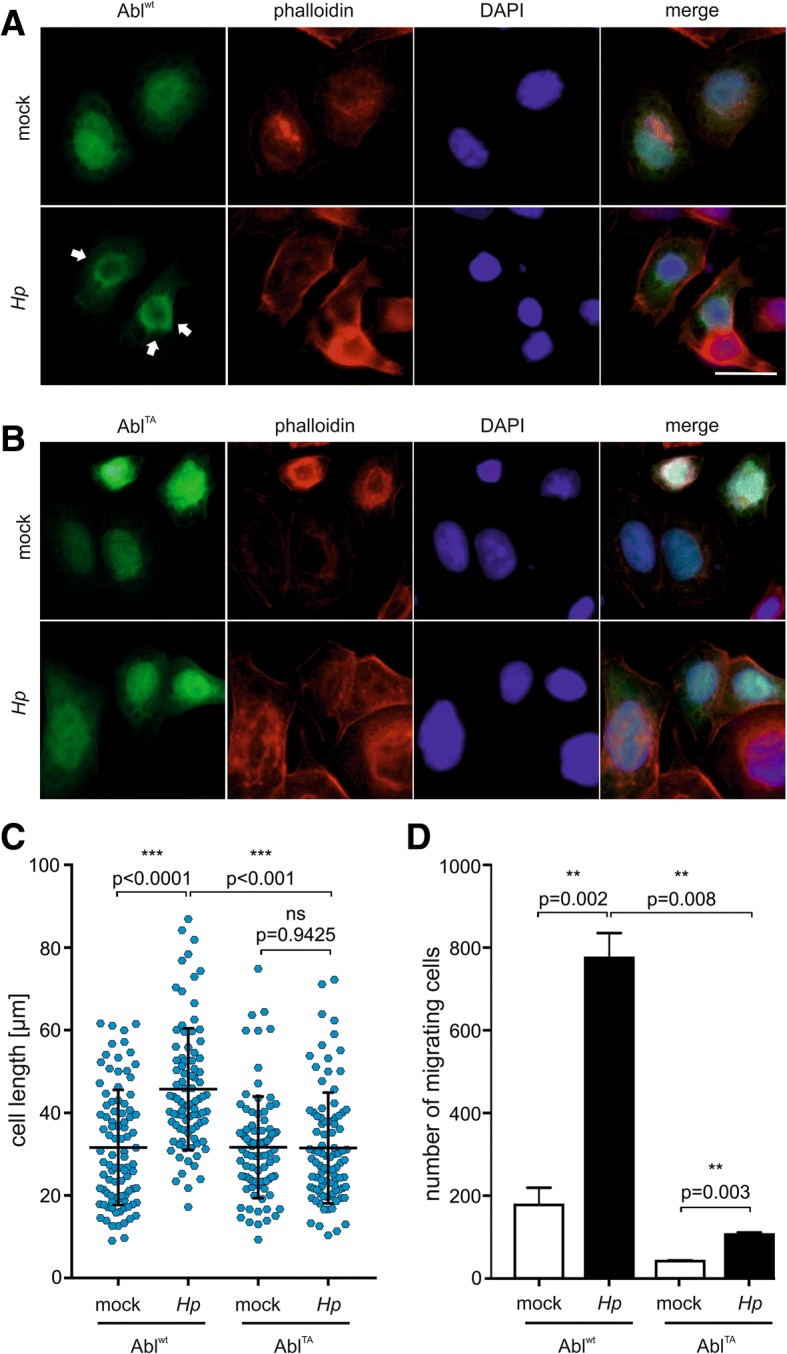
*Hp*-mediated pAbl^T735^ leads to cytoplasmic localization. AGS cells were transfected with Abl^wt^ **(a)** or Abl^TA^ **(b)**. Cells were left uninfected (mock) or infected with *Hp* for 4 h. Abl (green) and phalloidin (red) were merged with DAPI staining (blue). White arrows indicate perinuclear and cytoplasmic staining of c-Abl. Bar, 20 μm. **(c)** The lengths of approximately 100 transfected AGS cells expressing Abl^wt^ or Abl^TA^ were determined after infection with *Hp* for 4 h. **(d)** Stable AGS TAP-Abl^wt^ and TAP-Abl^TA^ cells were grown in transwell filters and left untreated (white bars) or infected with *Hp* (black bars) for 24 h

**Fig. 5:**
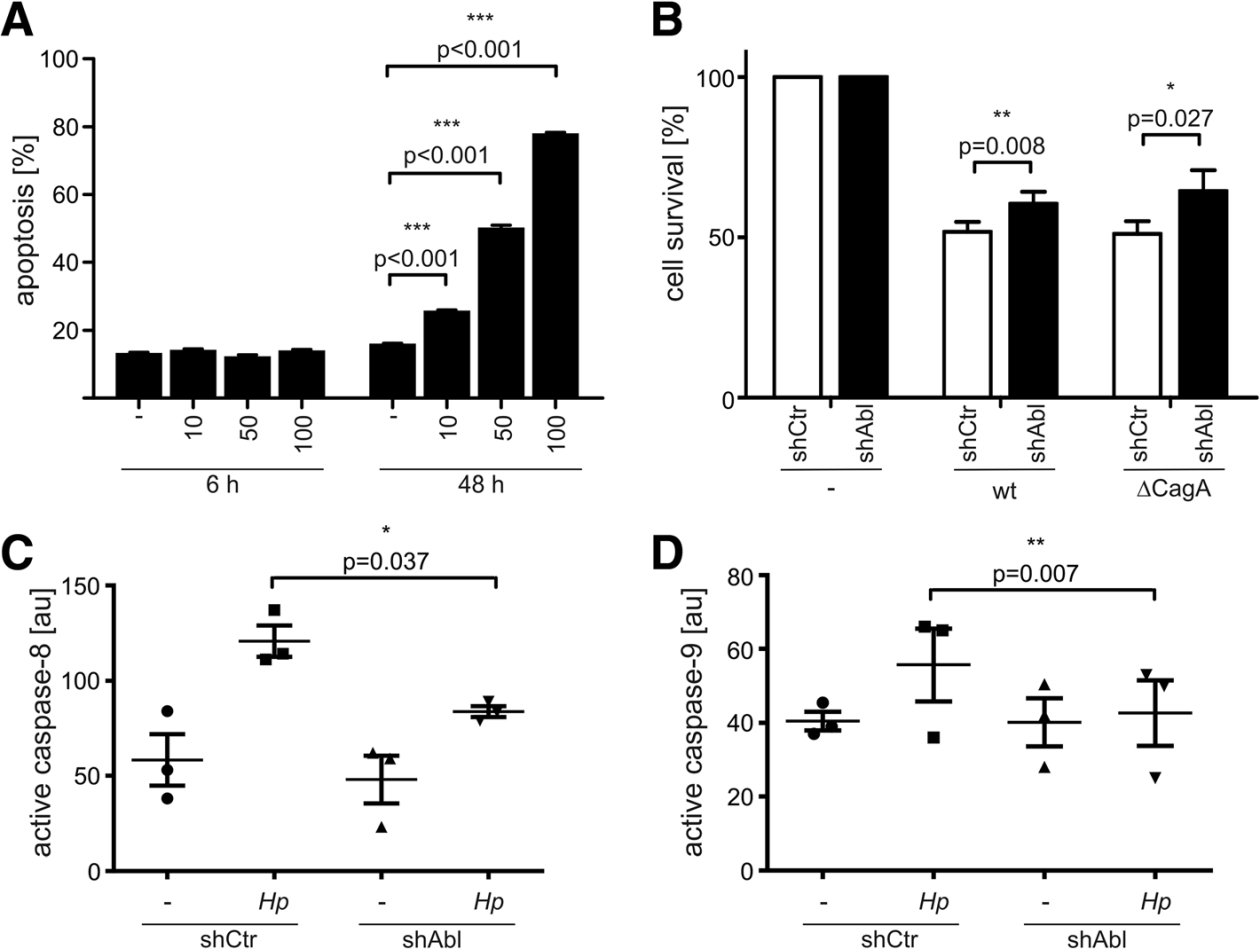
c-Abl downregulation reduced apoptosis. (**a**) AGS cells were left untreated or infected with *Hp* at the indicated MOIs and time periods. Percent apoptosis was calculated by analyzing combined Annexin single-positive and Annexin/propidium iodide-double positive cells. **(b, c, d)** Stable AGS cells transfected with control (shCtr) or c-Abl (shAbl) shRNA were left untreated or infected with *Hp* wt or a ∆*cagA* mutant at a MOI 20 for 48 h. Cell survival was determined using a MTT assay. **(b)** Equal protein amounts were subjected to human early apoptosis kit measurements and active caspase-8 **(c)** or caspase-9 **(d)** are shown

**Fig. 6:**
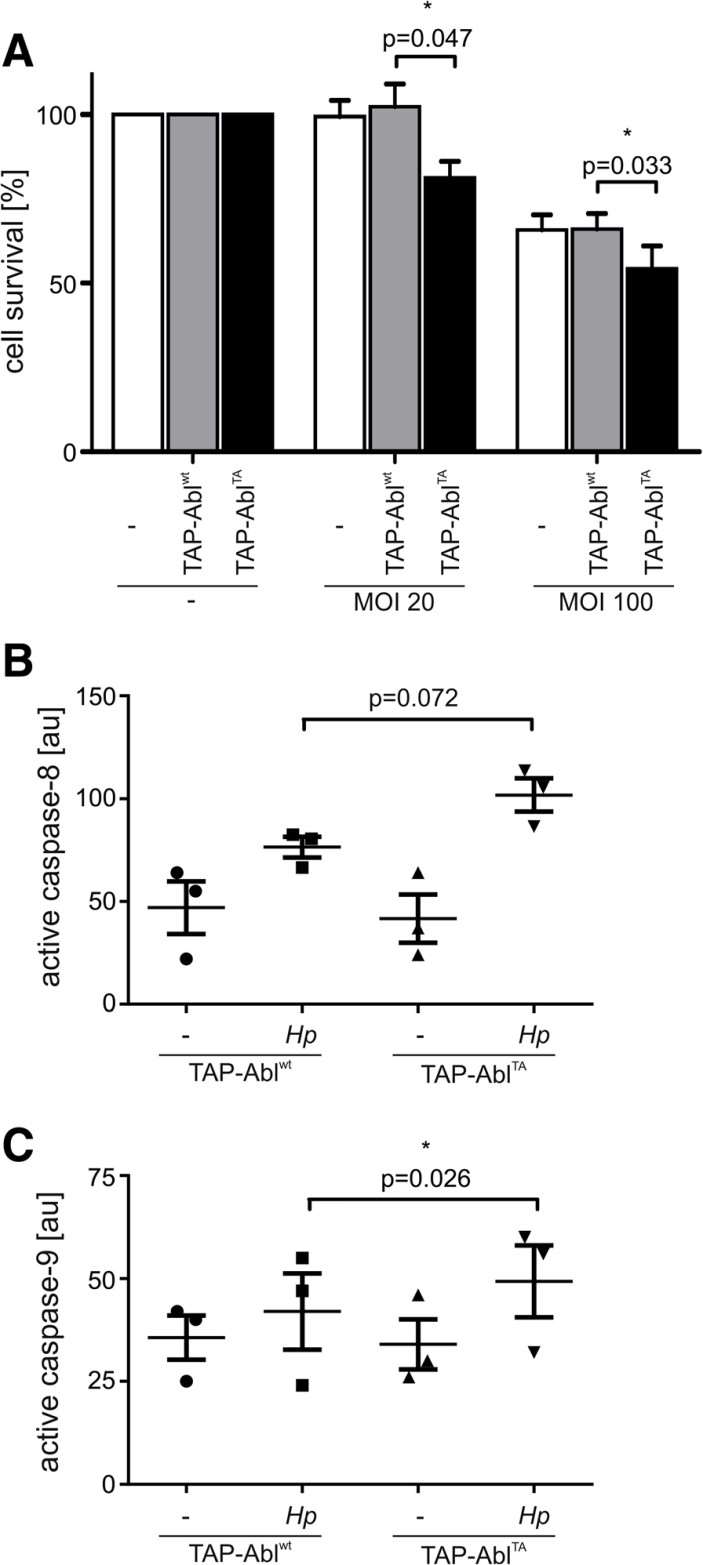
Phospho-resistant Abl^TA^ causes increased apoptosis. Stable TAP-Abl^wt^ or TAP-Abl^TA^ cells were left untreated or infected with *Hp* at indicated MOIs for 48 h. **(a)** Cell survival was determined using a MTT assay. Equal protein amounts were subjected to human early apoptosis kit measurements and active caspase-8 **(b)** or caspase-9 **(c)** are shown

14–3-3 interaction can cause cytoplasmic retention of c-Abl through binding to the phosphorylated threonine residue 735 and thus masking the NLS sequences. This interaction was shown to hinder the nuclear import of c-Abl in response to genotoxic or oxidative stress [32, 51]. Therefore, we analyzed the subcellular localization of c-Abl in *Hp*-infected cells. Immunofluorescence microscopy of AGS cells expressing c-Abl^wt^ (Fig. 4a) or c-Abl^TA^ (Fig. 4b) was performed. Both, non-infected c-Abl^wt^ and c-Abl^TA-^expressing cells showed no distinct localization and c-Abl was distributed in the cytoplasmic and nuclear compartment. This picture changed after infection with *Hp*. Here, c-Abl^wt^ showed nuclear exclusion and preferentially localized to perinuclear regions (Fig. 4a). In contrast, c-Abl^TA^ was mainly localized in the nuclei of infected cells (Fig. 4b). Quantification of nuclear c-Abl localization verified the retention of Abl^wt^ in the cytoplasm compared to Abl^TA^ (Additional file 4: Figure S3F). In *Hp* infections, the differential localization pattern was also accompanied by a reduced elongation phenotype, which was quantified by evaluating elongation of c-Abl-positive cells. Cells expressing c-Abl^wt^ exhibited the typical elongated cell morphology, which was drastically reduced in c-Abl^TA^-positive cells (Fig. 4c and Additional file 5: Figure S4B). This observation was further confirmed by the finding that c-Abl^TA^ expression led to a drastic decrease in *Hp*-induced cell migration as compared to c-Abl^wt^ expressing cells (Fig. 4d) implying that cytoplasmic localization of c-Abl is involved in actin cytoskeleton reorganization leading to cell elongation and motility. Importantly, the 14–3-3 antagonist BV02 clearly inhibited cell elongation (Additional file 4: Figure S3G), which underlines the significance of 14–3-3 binding in the regulation of cytoplasmic Abl functions in *Hp*-infected cells.

c-Abl is implicated in the DNA damage response by supporting G1 arrest and DNA repair and it also contributes to programmed cell death via p73- and presumably p63-dependent mechanisms [52, 53]. *Hp* is known to induce significant levels of apoptosis in vitro [54] and in vivo [55]. Therefore, we analyzed whether c-Abl contributes to the *Hp*-mediated apoptotic response. As expected, *Hp* induced apoptosis in a MOI-dependent manner (Fig. 5a). To analyze the role of c-Abl in cell survival, a c-Abl-deficient AGS cell line using stable shRNA-mediated RNA interference and a control cell line was employed [10] (Fig. 5b). In line with our hypothesis, c-Abl knock-down (shAbl) resulted in a significantly reduced apoptosis as compared to the control shRNA (shCtr) as monitored by MTT experiments (Fig. 5b) and apoptosis assays (Additional file 5: Figure S4E). Interestingly, this observation was also independent of CagA suggesting that the pro-apoptotic effect is mediated directly via c-Abl (Fig. 5b). Concomitantly with reduced cell death, we could also show decreased caspase-8 (Fig. 5c) and caspase-9 activation (Fig. 5d) in shAbl cells. Efficient knockdown of endogenous c-Abl expression in AGS cells was verified by Western blotting and the decrease in *Hp*-mediated cell elongation (Additional file 5: Figure S4C-D). Consequently, the observation that mutational disruption of the c-Abl/14–3-3 interaction resulted in increased nuclear localization of the Abl^TA^ mutant in *Hp* infected cells led to the question whether this also causes increased apoptosis. Ectopic TAP-Abl^TA^ expression rendered the cells more sensitive to apoptosis than TAP-Abl^wt^ after *Hp* infection (Fig. 6a), which was accompanied by an increased activation of caspase-8 (Fig. 6b) and caspase-9 (Fig. 6c) downstream of nuclear c-Abl processes [56]. These data support our conclusion that increased nuclear localization of Abl^TA^ correlates with increased apoptosis, while cell migration is inhibited.

### Increased c-Abl expression and phosphorylation in *Hp* pathologies in vivo

**Fig. 7:**
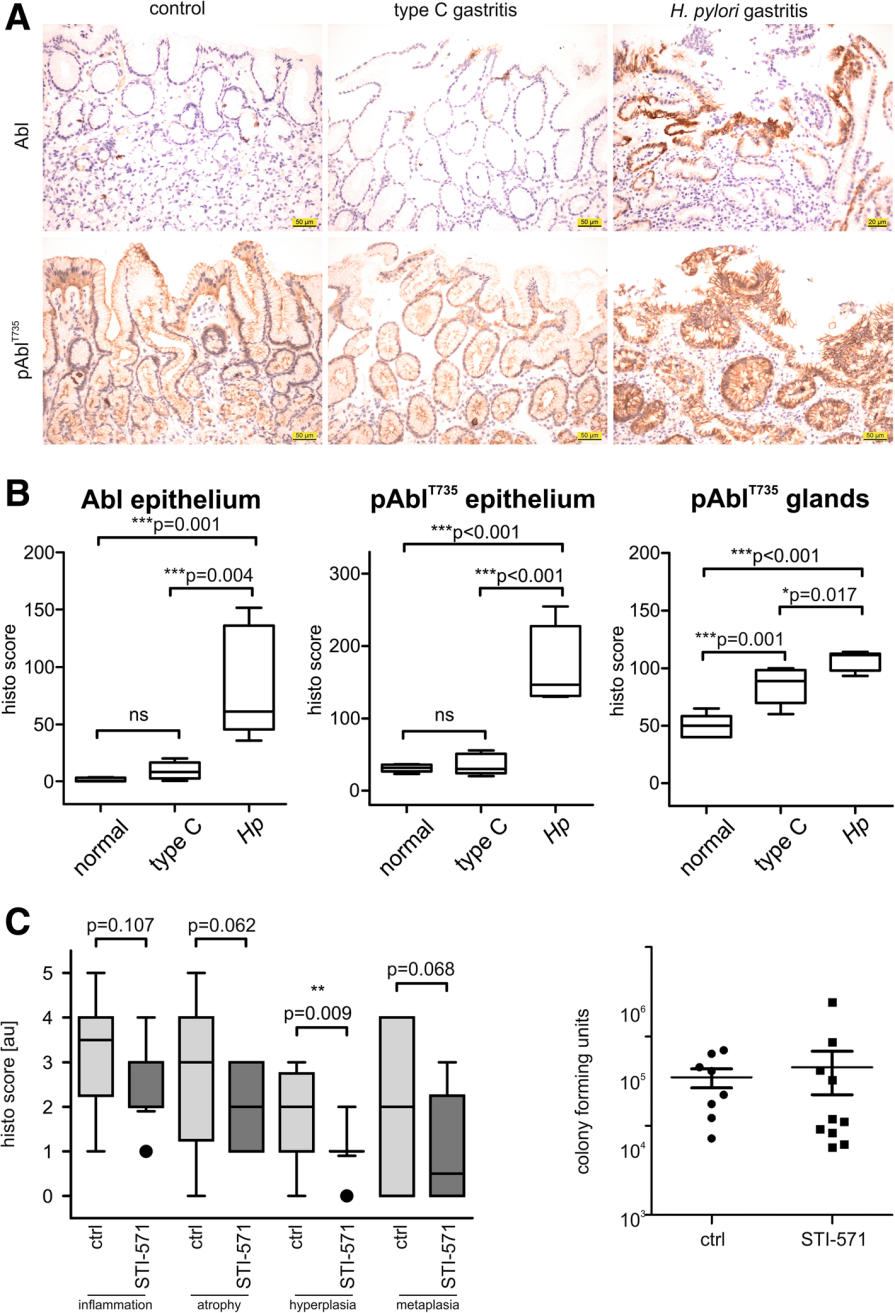
*Hp* induces pAbl^T735^ phosphorylation and activation to promote pathogenesis in vivo. (**a**) Tissue samples from healthy (control), type-C gastritis and *Hp*-mediated gastritis patients were stained with hematoxylin and antibodies recognizing c-Abl or pAbl^T735^. **(b)** Histology scores of the analyzed sections were evaluated for Abl and pAbl^T735^ in the epithelium and gastric glands. **(c)** C57BL/6 mice were infected with PMSS1 for two months and remained untreated (ctrl) or were supplied with STI-571. Gastric tissue sections were analyzed for chronic inflammation, gastric atrophy, intestinal metaplasia and mucus pit cell/epithelial hyperplasia (left panel). Mice were analyzed for *Hp* colonization using *cfu* assays (right panel). Box blots show median, 25th and 75th percentile (box) and 5th and 95th percentile (whiskers)

To investigate whether *Hp* exhibited similar effects on c-Abl expression and phosphorylation in vivo, we analyzed gastric tissue samples obtained from patients diagnosed with type C (chemically induced) gastritis or *Hp*-associated B gastritis and compared them to healthy controls. *Hp*-associated gastritis specimens displayed a considerable tissue infiltrate with lymphocytes. Concomitantly, a strong increase in the expression of c-Abl was observed. Importantly, an increased phosphorylation of pAbl^T735^ was detected in the *Hp*-positive samples, but not in type C gastritis (Fig. 7a). Histological scoring of c-Abl and pAbl^T735^ resulted in a significant association between *Hp* infections, enhanced c-Abl expression and phosphorylation of pAbl^T735^ in the gastric epithelium and gastric glands (Fig. 7b).

Next, we addressed the role of c-Abl in *Hp-*associated pathogenesis using a murine infection model. The kinase activity of c-Abl was pharmacologically inhibited using Gleevec (STI-571), which did not induce gastric pathologies in uninfected mice [45]. C57BL/6 mice were infected with *Hp* for two months and were analyzed for successful colonization (Fig. 7c, right panel) and disease parameters were quantified by histology (Fig. 7c, left panel and Additional file 6: Figure S5). Despite the long-term treatment with Gleevec, we observed a similar level of colonization and inflammation in both groups. Apart from inflammation all disease parameters were decreased in the Gleevec-treated animals. In particular reduction of hyperplasia was highly significant (*p* = 0.0091) (Fig. 7c and Additional file 6: Figure S5). This further underlines the critical contribution of the c-Abl signaling axis in the onset and progression of *Hp*-induced pathology.

## Discussion

**Fig. 8:**
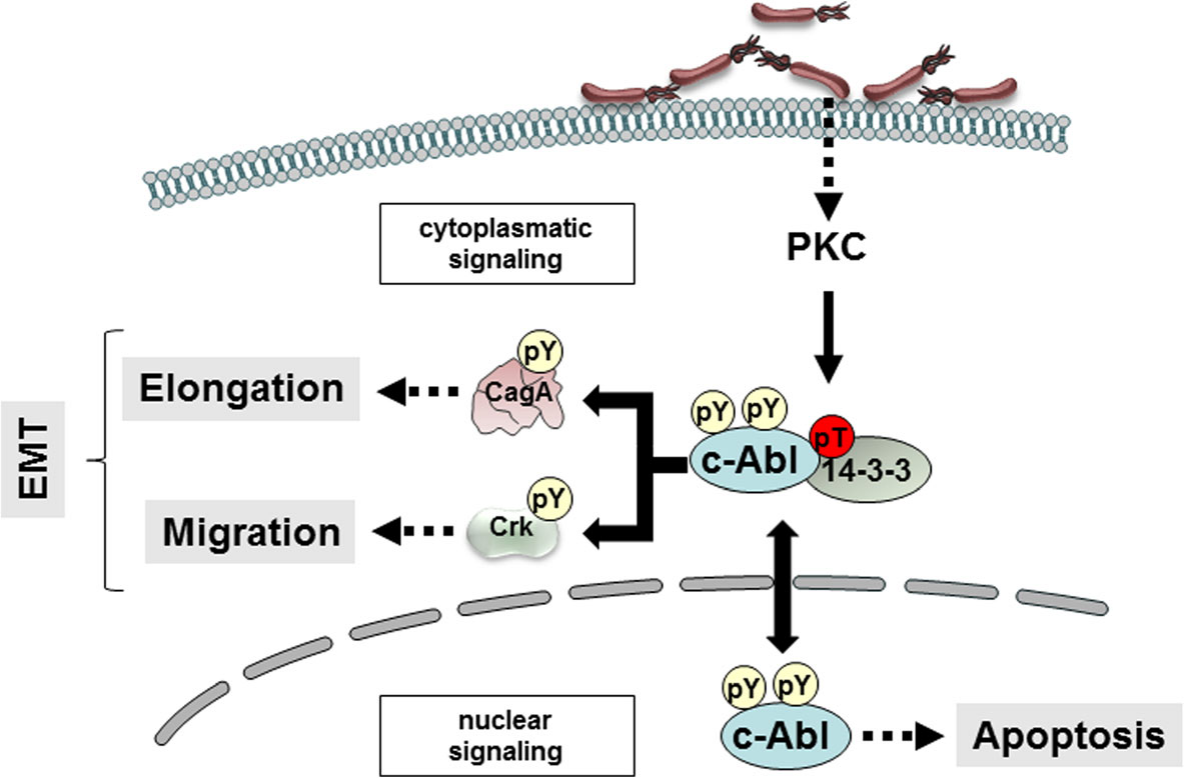
Model of c-Abl regulation in *Hp*-infected cells. *Hp* induces tyrosine and threonine phosphorylation of c-Abl. Tyrosine phosphorylation leads to c-Abl kinase activation. Threonine 735 phosphorylation is directly mediated by PKC and forces cytoplasmic localization of c-Abl via binding to 14–3-3 proteins. Cytoplasmic retention of activated c-Abl promotes cell elongation and migration contributing to the EMT-like phenotype and attenuates apoptotic functions in the nucleus

The implication of c-Abl in *Hp* pathogenesis is well established and a multifaceted deregulation of host cell signaling has been demonstrated in *Hp*-infected cells in vitro and animal models [9, 10, 45]. Importantly, c-Abl is responsible for sustained CagA phosphorylation after inactivation of Src kinases in gastric epithelial cells and significantly contributes to cytoskeletal rearrangement and cell motility resulting in an EMT-like scatter-phenotype [9, 10]. However, little is known about the regulation of c-Abl in this complex network of signaling cascades. Here, we report a novel mechanism of c-Abl regulation in *Hp*-infected cells and demonstrate that pAbl^T735^ functions as decisive switch for the subcellular localization of c-Abl. This reinforces cytoplasmic processes facilitating cell migration and elongation, while pro-apoptotic effects in the nucleus are prevented (Fig. 8).

The nuclear functions of c-Abl have been intensively investigated and include DNA damage response, inhibition of cell growth and apoptosis [57]. In our experiments, we found that nuclear localization of c-Abl^TA^ increases apoptosis and caspase activation. These data are supported by findings that nuclear c-Abl promotes apoptosis in a p73- and p63-dependent manner, and eventually activates the intrinsic apoptosis pathway and initiator caspases in a feedback loop [52, 56, 58]. The cytoplasmic functions of c-Abl are less well defined. Interestingly, in *Hp*-infected cells, endogenous c-Abl mainly localizes in the cytoplasm and was also detected in focal adhesion contacts [10] suggesting that deregulated c-Abl promotes cell elongation and migration. A similar subcellular distribution was observed in cells ectopically expressing c-Abl and we identified pAbl^T735^ phosphorylation as a critical regulator of its subcellular localization (Fig. 8). The discovery of *Hp*-mediated pAbl^T735^ adds an important novel aspect in the c-Abl-mediated regulation of cytoplasmic and nuclear processes in *Hp*-infected epithelial cells. Mechanistically, we showed that 14–3-3 binds to c-Abl in a phospho-threonine 735-dependent manner and thus forces cytoplasmic localization [31]. Hence, activated c-Abl in the cytoplasm potentiates cytoskeletal rearrangements, which are implicated in cell elongation and migration, while nuclear depletion attenuated apoptosis in response to *Hp* (Fig. 8).

Recent publications suggested that TTK/Mps1 can directly phosphorylate pAbl^T735^ upon oxidative stress [32]. Instead of TTK, we identified *Hp-*activated PKCs as novel kinases that directly phosphorylate pAbl^T735^ (Fig. 8). *Hp* induces a wide range of conventional, novel and atypical PKCs; therefore, PKCs are interesting targets per se in *Hp* pathogenesis since they are involved in proliferation, cell scattering and cellular invasion [42, 59]. Hence, the identification of c-Abl as a novel PKC substrate might represent a missing link between PKC activation and the aforementioned cellular responses. pAbl^T735^ was induced independently of CagA, but required a functional T4SS. This is in agreement with a study of Sokolova et al. showing that PKC activation is mediated by T4SS-dependent and T4SS-independent factors [59]. This points to the hypothesis that CagL could trigger pAbl^T735^ possibly via β1-integrin activation, since it has been suggested that c-Abl activation and its nuclear export is regulated via integrin signaling [60]. In fact, tyrosine phosphorylation of c-Abl is CagA- and CagL-dependent, and both factors were shown to activate β1-integrin signaling [15, 36]. However, in our experiments CagL was not solely responsible for pAbl^T735^, but we detected a significant influence of the newly identified T4SS effector βHBP. βHBP is delivered independently of CagA into host cells where it activates the ALPK1-TIFAsome-NF-kB pathway [37, 49]. Therefore, we propose a model that βHBP links the T4SS to the anti-apoptotic function of cytoplasmic c-Abl and that different signal transduction pathways are involved to control phospho-tyrosine-dependent c-Abl activity and phospho-threonine-dependent subcellular localization.

Importantly, c-Abl is a disease-promoting factor in *Hp-*associated gastric pathologies in vivo. First, a significant increase in c-Abl levels was observed in the gastric epithelium and glands in patients suffering from *Hp*-associated gastritis, but not in type-C gastritis. The elevated expression levels were accompanied by a drastic phosphorylation of pAbl^T735^ and a preferential cytoplasmic localization in analyzed specimens. Second, in murine infection models we demonstrated that continuous inhibition of c-Abl kinase activity alleviated *Hp-*induced gastric disease parameters, particularly mucus pit cell/epithelial hyperplasia. Together, the in vivo experiments indicate that the observed effects of c-Abl are crucially involved in lasting and chronic *Hp* infections, which exceed the time frames typically covered in in vitro settings.

## Conclusions

In previous studies we have shown that *Hp* activates c-Abl kinase to maintain CagA phosphorylation [9, 10]. The finding that *Hp* infections do not only induce c-Abl kinase activity, but also forces cytoplasmic localization of the activated kinase, where it promotes cell migration and elongation and actively prevents apoptosis (Fig. 8) adds an important new aspect to the complex mechanism of *Hp*-mediated carcinogenesis.

## Additional file

Additional file 1: Supplementary Information. (DOCX 32 kb)

Additional file 2: **Figure S1.** c-Abl threonine phosphorylation in AGS, MKN28 and MCF-7 cells. (A) AGS cells were transfected with pSGT-Abl^wt^ and either left untreated or infected with *Hp* wt for the indicated periods of time. Levels of pAbl^T735^ (white bars) and total Abl (black bars) were quantified by blot densitometry and normalized to GAPDH. Fold changes compared to uninfected cells are shown. (B) MKN28 and (C) MCF-7 cells were transfected with pSGT-Abl and either left untreated or infected with *Hp* for the indicated periods of time. Levels of pAbl^T735^, total c-Abl and GAPDH are shown. (D) Lysates of uninfected (−) and *Hp*-infected (+) MKN28 cells were subjected to immunoprecipitation (IP) using a specific c-Abl antibody. Lysates before IP (pre IP), the precipitates (IP) and lysates after IP (post IP) were analyzed by Western blotting to detect pAbl^T735^ and c-Abl. (E) Transfected AGS cells were infected with several Western (P12, P1, Hp26695 and B8) and East Asian *Hp* isolates (42GX and 48GX) and analyzed by Western blotting to detect pAbl^T735^, c-Abl and GAPDH. (DOCX 180 kb)

Additional file 3: **Figure S2.** Tyrosine phosphorylation, but not threonine phosphorylation of c-Abl depends on CagL. (A) AGS cells were transfected with pSGT-Abl^wt^ and remained uninfected or were infected with isogenic *Hp* wt, ΔCagL, or ΔCagL/CagL strains for 6 h. Whole cell lysates were subjected to Western blotting to analyze pAbl^T735^, pAbl^Y245^ and pAbl^Y412^. c-Abl and β-actin were shown as loading controls. Infections were further analyzed for pCagA and CagA. (B) Quantification of pAbl^T735^, pAbl^Y245^ and pAbl^Y412^ was performed by Western blot densitometry, which was normalized to corresponding β-actin levels. Graphs show mean ± SD of three independent experiments. (C) Cells were infected with *Hp* wt, ΔRfaE or ΔPAI. pAbl^T735^, Abl^Y245^, pCagA, CagA and GAPDH were detected using specific antibodies. (DOCX 2290 kb)

Additional file 4: **Figure S3.** Differential phosphorylation patterns in c-Abl mutants. (A) AGS cell were transfected with pSGT-Abl^wt^, pSGT-Abl^TA^, pSGT-Abl^PP^, pSGT-Abl^KD^, pSGT-Abl^Y245F^, pSGT-cAbl^Y412F^, or empty vector (ut) and either left untreated, infected with *Hp* wt or stimulated with H_2_O_2_/vanadate (H/V, left panel) or PMA (right panel) for 6 h. Whole cell lysates were analyzed by Western blotting for pAbl^T735^, pAbl^Y245^ or pAbl^Y412^, pCagA, CagA, GAPDH and β-actin. Quantification of pAbl^T735^ (B) pAbl^Y245^ (C) and pAbl^Y412^ (D) were performed by blot densitometry and normalized to the corresponding β-actin levels. Graphs present mean ± SD of three independent experiments. (E) Transfected AGS cells were pretreated with 10 μM STI-571 and infected with *Hp* for 6 h as indicated. Whole cell lysates were analyzed by Western blotting for pAbl^T735^, pAbl^Y245^, Abl and GAPDH. (F) AGS cells were transfected with pSGT-Abl^wt^ or pSGT-Abl^TA^ and then infected with *Hp* for 4 h. Nuclear and cytoplasmic localization was quantified from four independent experiments. (G) AGS stably transfected with pNTAP Abl^wt^ were pretreated with a 14–3-3 inhibitor (BV02) or vehicle control (DMSO) and infected with *Hp* for 8 h. Cell elongation was determined by measuring the largest cell diameter of individual cells from three independent experiments. (DOCX 310 kb)

Additional file 5: **Figure S4.** Generation of stable AGS cell lines. (A) Untreated AGS cells and AGS cells transfected with TAP-Abl^wt^ or TAP-Abl^TA^ were either left untreated (mock) or infected with *Hp* at a MOI 100 for 6 h and analyzed by Western blot for pAbl^T735^ and c-Abl. β-actin served as loading control. (B) Untreated AGS cells and AGS cells expressing TAP-Abl^wt^ or TAP-Abl^TA^ were either left untreated (mock) or infected with *Hp* at a MOI 100. The scattering phenotype was documented using phase contrast microscopy. (C) Untreated AGS cells and AGS cells stably transfected with control shRNA (shCtrl) or c-Abl shRNA (shAbl) were lysed and analyzed by Western blotting for c-Abl and GAPDH expression (D) AGS cells stably transfected with control shRNA (shCtrl) or c-Abl shRNA (shAbl) were either left untreated (mock) or infected with *Hp* at a MOI 100 for 6 h. Scattering phenotype was documented using phase contrast microscopy. (E) AGS cells stably transfected with control (shCtr) or Abl shRNA (shAbl) were left untreated (−) or infected with *Hp* wt for 48 h. Percent apoptosis was calculated by analyzing annexin single-positive and annexin/7AAD positive cells. (DOCX 276 kb)

Additional file 6: **Figure S5.** Gleevec decreases *Hp* pathology. C57BL/6 mice were infected with *Hp* PMSS1 for two months, were supplied with STI-571 or remained untreated (control). Representative sections of the gastric tissues are shown. (DOCX 261 kb)

## Abbreviations

CagA: Cytotoxin-associated gene A
*Hp*: *Helicobacter pylori*
PKC: Protein kinase C
βHBP: β-glycero-β-D-manno-heptose-1,7-bisphosphate
