# Supplementary material for: Helicobacter pylori-controlled c-Abl localization promotes cell migration and limits apoptosis
Source: Cell Commun Signal. 2019 Jan 31;17:10. doi: 10.1186/s12964-019-0323-9 (PMC6357398; doi:10.1186/s12964-019-0323-9)
Supplement: Supplementary file 1 — Supplementary Information. (DOCX 32 kb) [file 12964_2019_323_MOESM1_ESM.docx]

**Supplementary Methods**

*PKC siRNA.* PKC siRNA (h) (sc-29449, Santa Cruz) is a pool of five different siRNA duplexes. sc-29449A targets eta NM_006255 and epsilon NM_005400: sense ACCAAGCAGAAGACCAACAtt, antisense: UGUUGGUCUUCUGCUUGGUtt; sc-29449B targets beta NM_212535, alpha NM_002737, and gamma NM_002739: sense: CACUGCACCGACUUCAUCUtt, antisense: AGAUGAAGUCGGUGCAGUGtt; sc-29449C targets mu NM_002742, and nu NM_005813: sense: UCAGUCCAUCAACAAGCAAtt, antisense: UUGCUUGUUGAUGGACUGAtt; sc-29449D targets delta NM_212539 and theta NM_006257: sense: GGGAUGUGCAAAGAGAACAtt, antisense: UGUUCUCUUUGCACAUCCCtt; sc-29449E targets zeta NM_002744 and iota NM_002740: sense: CAGAGAAGCACGUGUUUGAtt, antisense: UCAAACACGUGCUUCUCUGtt.

*Mouse colonization experiments.* One to two longitudinal sections per mouse spanning the length of the stomach from the forestomach/corpus junction to the antrum/duodenum junction were scored with regard to four histopathological parameters (chronic inflammation, gastric atrophy, intestinal metaplasia, mucus pit cell/epithelial hyperplasia), based on the features described in the updated Sydney classification (1). We attributed scores on a scale of 0-6 as proposed by Chen *et al.* (2). Specifically, the definition of scores was as follows for the four parameters evaluated. Chronic inflammation: 0, none; 1, some infiltrates; 2, mild (few aggregates in submucosa and mucosa); 3, moderate (several aggregates in submucosa and mucosa); 4, marked (many big aggregates in submucosa and mucosa); 5, nearly the entire mucosa contains a dense infiltrate; 6, entire mucosa contains a dense infiltrate. Atrophy: 0, none; 1, foci where a few gastric glands are lost or replaced; 2, small areas in which gastric glands have disappeared or been replaced; 3, <25% of gastric glands lost or replaced; 4, 25-50% of gastric glands lost or replaced; 5, >50% of gastric glands lost or replaced; 6, only a few small areas of gastric differentiated glands remaining. Intestinal metaplasia: 0, none; 1, only one crypt replaced by intestinal epithelium (i.e.); 2, one focal area (1-4 crypts) replaced; 3, two separate foci with metaplasia; 4, multiple foci; 5, >50% of gastric epithelium replaced by i.e.; 6, only a few small areas of gastric epithelium are not replaced by i.e. Hyperplasia: 0,  none; 1, single glands (next to infiltrate); 2, one focal area /1-4 crypts (mild); 3; 1-3 foci; 4, multiple foci; 5, > 50% of glands affected; 6, only few small non-hyperplasic areas.

*Immunohistochemistry*. 3 µm thick, archival FFPE sections were deparaffinized with xylene, dehydrated, followed by heat-induced epitope retrieval (HIER) at 98°C for 40 minutes in antigen retrieval buffer pH 6 (**Agilent Technologies**, Austria). Endogenous peroxidase blocking was carried out for 10 minutes with 3% H_2_O_2_ in absolute methanol and normal serum was applied. Primary antibodies (c-Abl clone 8E9, Becton Dickinson, Austria and anti-pAbl^T735^, **New England Biolabs,** Germany) and detection reagents were incubated at room temperature for 30 minutes and after several washes detection was performed using Envision detection system, followed by chromogenic visualisation with diaminobenzidine (DAB). Nuclear counterstaining was performed with hematoxylin. Staining intensity (0 - 3) and extent (0% - 100% cells) of the antibody stainings were assessed by an experienced pathologist for the gastric specimens, separately for the surface epithelium as well as for the cells of the gastric glands by evaluating 100 cells. Semi-quantitative immunohistochemistry scores were calculated by multiplication of the intensity and extensity yielding scores between 0 and 300 as already applied (3, 4) and established earlier (5).

**Supplementary Literature**

1. Dixon MF, Genta RM, Yardley JH, Correa P. Classification and grading of gastritis. The updated Sydney System. International Workshop on the Histopathology of Gastritis, Houston 1994. The American journal of surgical pathology. 1996;20(10):1161-81.

2. Chen XY, van der Hulst RW, Bruno MJ, van der Ende A, Xiao SD, Tytgat GN, et al. Interobserver variation in the histopathological scoring of Helicobacter pylori related gastritis. Journal of clinical pathology. 1999;52(8):612-5.

3. Urbas R, Mayr C, Klieser E, Fuereder J, Bach D, Stattner S, et al. Relevance of MicroRNA200 Family and MicroRNA205 for Epithelial to Mesenchymal Transition and Clinical Outcome in Biliary Tract Cancer Patients. International journal of molecular sciences. 2016;17(12).

4. Kiesslich T, Alinger B, Wolkersdorfer GW, Ocker M, Neureiter D, Berr F. Active Wnt signalling is associated with low differentiation and high proliferation in human biliary tract cancer in vitro and in vivo and is sensitive to pharmacological inhibition. International journal of oncology. 2010;36(1):49-58.

5. Detre S, Saclani Jotti G, Dowsett M. A "quickscore" method for immunohistochemical semiquantitation: validation for oestrogen receptor in breast carcinomas. Journal of clinical pathology. 1995;48(9):876-8.
