# Supplementary material for: Helicobacter pylori-controlled c-Abl localization promotes cell migration and limits apoptosis
Source: Cell Commun Signal. 2019 Jan 31;17:10. doi: 10.1186/s12964-019-0323-9 (PMC6357398; doi:10.1186/s12964-019-0323-9)
Supplement: Supplementary file 2 — Figure S1. c-Abl threonine phosphorylation in AGS, MKN28 and MCF-7 cells. (A) AGS cells were transfected with pSGT-Ablwt and either left untreated or infected with Hp wt for the indicated periods of time. Levels of pAblT735 (white bars) and total Abl (black bars) were quantified by blot densitometry and normalized to GAPDH. Fold changes compared to uninfected cells are shown. (B) MKN28 and (C) MCF-7 cells were transfected with pSGT-Abl and either left untreated or infected with Hp for the indicated periods of time. Levels of pAblT735, total c-Abl and GAPDH are shown. (D) Lysates of uninfected (−) and Hp-infected (+) MKN28 cells were subjected to immunoprecipitation (IP) using a specific c-Abl antibody. Lysates before IP (pre IP), the precipitates (IP) and lysates after IP (post IP) were analyzed by Western blotting to detect pAblT735 and c-Abl. (E) Transfected AGS cells were infected with several Western (P12, P1, Hp26695 and B8) and East Asian Hp isolates (42GX and 48GX) and analyzed by Western blotting to detect pAblT735, c-Abl and GAPDH. (DOCX 180 kb) [file 12964_2019_323_MOESM2_ESM.docx]

**
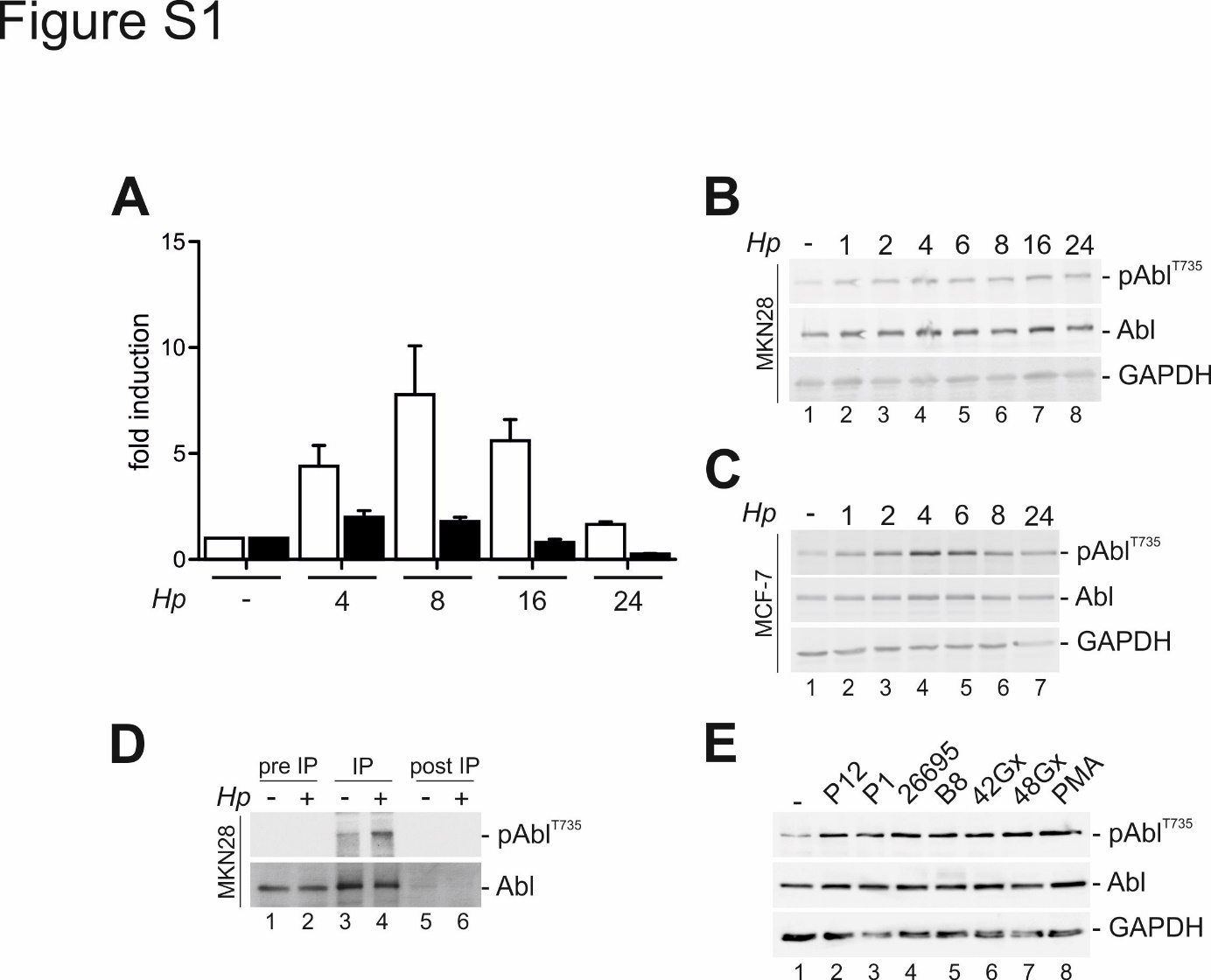
**

**Figure S1. c-Abl threonine phosphorylation in AGS, MKN28 and MCF-7 cells**. **(A)** AGS cells were transfected with pSGT-Abl^wt^ and either left untreated or infected with *Hp* wt for the indicated periods of time. Levels of pAbl^T735^ (white bars) and total Abl (black bars) were quantified by blot densitometry and normalized to GAPDH. Fold changes compared to uninfected cells are shown. **(B)** MKN28 and **(C)** MCF-7 cells were transfected with pSGT-Abl and either left untreated or infected with *Hp* for the indicated periods of time. Levels of pAbl^T735^, total c-Abl and GAPDH are shown. **(D)** Lysates of uninfected (-) and *Hp*-infected (+) MKN28 cells were subjected to immunoprecipitation (IP) using a specific c-Abl antibody. Lysates before IP (pre IP), the precipitates (IP) and lysates after IP (post IP) were analyzed by Western blotting to detect pAbl^T735^ and c-Abl. **(E)** Transfected AGS cells were infected with several Western (P12, P1, Hp26695 and B8) and East Asian *Hp* isolates (42GX and 48GX) and analyzed by Western blotting to detect pAbl^T735^, c-Abl and GAPDH.
