# Supplementary material for: Helicobacter pylori-controlled c-Abl localization promotes cell migration and limits apoptosis
Source: Cell Commun Signal. 2019 Jan 31;17:10. doi: 10.1186/s12964-019-0323-9 (PMC6357398; doi:10.1186/s12964-019-0323-9)
Supplement: Supplementary file 3 — Figure S2. Tyrosine phosphorylation, but not threonine phosphorylation of c-Abl depends on CagL. (A) AGS cells were transfected with pSGT-Ablwt and remained uninfected or were infected with isogenic Hp wt, ΔCagL, or ΔCagL/CagL strains for 6 h. Whole cell lysates were subjected to Western blotting to analyze pAblT735, pAblY245 and pAblY412. c-Abl and β-actin were shown as loading controls. Infections were further analyzed for pCagA and CagA. (B) Quantification of pAblT735, pAblY245 and pAblY412 was performed by Western blot densitometry, which was normalized to corresponding β-actin levels. Graphs show mean ± SD of three independent experiments. (C) Cells were infected with Hp wt, ΔRfaE or ΔPAI. pAblT735, AblY245, pCagA, CagA and GAPDH were detected using specific antibodies. (DOCX 2290 kb) [file 12964_2019_323_MOESM3_ESM.docx]

**Figure S2. Tyrosine phosphorylation, but not threonine phosphorylation of c-Abl depends on CagL**. **(A)** AGS cells were transfected with pSGT-Abl^wt^ and remained uninfected or were infected with isogenic *Hp* wt, ΔCagL, or ΔCagL/CagL strains for 6 hours. Whole cell lysates were subjected to Western blotting to analyze pAbl^T735^, pAbl^Y245^ and pAbl^Y412^. c-Abl and β-actin were shown as loading controls. Infections were further analyzed for pCagA and CagA. **(B)** Quantification of pAbl^T735^, pAbl^Y245^ and pAbl^Y412^ was performed by Western blot densitometry, which was normalized to corresponding β-actin levels. Graphs show mean ± SD of three independent experiments. **(C)** Cells were infected with *Hp* wt, ΔRfaE or ΔPAI. pAbl^T735^, Abl^Y245^, pCagA, CagA and GAPDH were detected using specific antibodies.
