# Supplementary material for: Helicobacter pylori-controlled c-Abl localization promotes cell migration and limits apoptosis
Source: Cell Commun Signal. 2019 Jan 31;17:10. doi: 10.1186/s12964-019-0323-9 (PMC6357398; doi:10.1186/s12964-019-0323-9)
Supplement: Supplementary file 4 — Figure S3. Differential phosphorylation patterns in c-Abl mutants. (A) AGS cell were transfected with pSGT-Ablwt, pSGT-AblTA, pSGT-AblPP, pSGT-AblKD, pSGT-AblY245F, pSGT-cAblY412F, or empty vector (ut) and either left untreated, infected with Hp wt or stimulated with H2O2/vanadate (H/V, left panel) or PMA (right panel) for 6 h. Whole cell lysates were analyzed by Western blotting for pAblT735, pAblY245 or pAblY412, pCagA, CagA, GAPDH and β-actin. Quantification of pAblT735 (B) pAblY245 (C) and pAblY412 (D) were performed by blot densitometry and normalized to the corresponding β-actin levels. Graphs present mean ± SD of three independent experiments. (E) Transfected AGS cells were pretreated with 10 μM STI-571 and infected with Hp for 6 h as indicated. Whole cell lysates were analyzed by Western blotting for pAblT735, pAblY245, Abl and GAPDH. (F) AGS cells were transfected with pSGT-Ablwt or pSGT-AblTA and then infected with Hp for 4 h. Nuclear and cytoplasmic localization was quantified from four independent experiments. (G) AGS stably transfected with pNTAP Ablwt were pretreated with a 14–3-3 inhibitor (BV02) or vehicle control (DMSO) and infected with Hp for 8 h. Cell elongation was determined by measuring the largest cell diameter of individual cells from three independent experiments. (DOCX 310 kb) [file 12964_2019_323_MOESM4_ESM.docx]

**Figure S3**


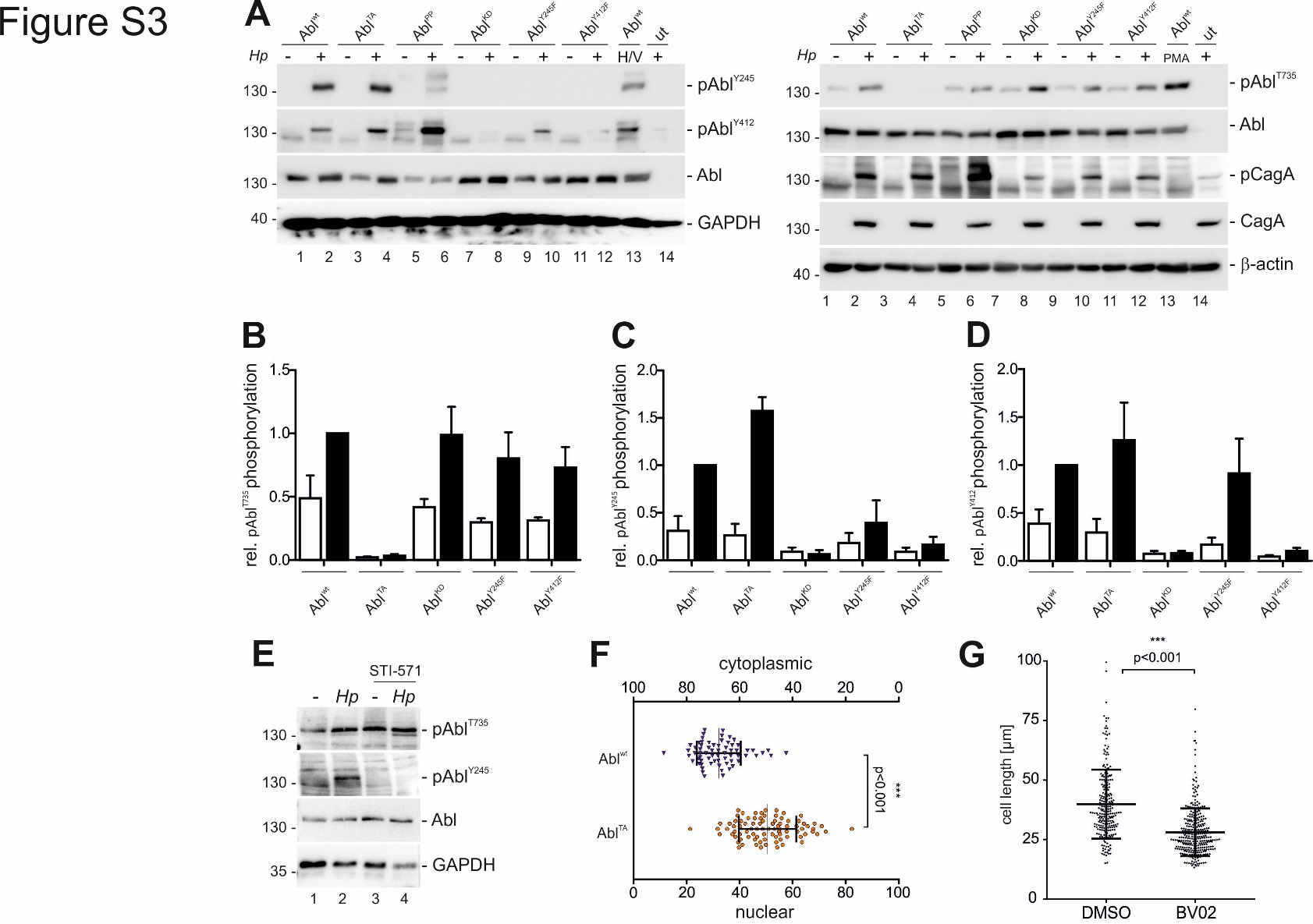


**Figure S3.** **Differential phosphorylation patterns in c-Abl mutants.** **(A)** AGS cell were transfected with pSGT-Abl^wt^, pSGT-Abl^TA^, pSGT-Abl^PP^, pSGT-Abl^KD^, pSGT-Abl^Y245F^, pSGT-cAbl^Y412F^, or empty vector (ut) and either left untreated, infected with *Hp* wt or stimulated with H_2_O_2_/vanadate (H/V, left panel) or PMA (right panel) for 6 hours. Whole cell lysates were analyzed by Western blotting for pAbl^T735^, pAbl^Y245^ or pAbl^Y412^, pCagA, CagA, GAPDH and β-actin. Quantification of pAbl^T735^ **(B)** pAbl^Y245^ **(C)** and pAbl^Y412^ **(D)** were performed by blot densitometry and normalized to the corresponding β-actin levels. Graphs present mean ± SD of three independent experiments. **(E)** Transfected AGS cells were pretreated with 10 µM STI-571 and infected with *Hp* for 6 hours as indicated. Whole cell lysates were analyzed by Western blotting for pAbl^T735^, pAbl^Y245^, Abl and GAPDH. **(F)** AGS cells were transfected with pSGT-Abl^wt^ or pSGT-Abl^TA^ and then infected with *Hp* for 4 hours. Nuclear and cytoplasmic localization was quantified from four independent experiments. **(G)** AGS stably transfected with pNTAP Abl^wt^ were pretreated with a 14-3-3 inhibitor (BV02) or vehicle control (DMSO) and infected with *Hp* for 8 hours. Cell elongation was determined by measuring the largest cell diameter of individual cells from three independent experiments.
