# Supplementary material for: Helicobacter pylori-controlled c-Abl localization promotes cell migration and limits apoptosis
Source: Cell Commun Signal. 2019 Jan 31;17:10. doi: 10.1186/s12964-019-0323-9 (PMC6357398; doi:10.1186/s12964-019-0323-9)
Supplement: Supplementary file 5 — Figure S4. Generation of stable AGS cell lines. (A) Untreated AGS cells and AGS cells transfected with TAP-Ablwt or TAP-AblTA were either left untreated (mock) or infected with Hp at a MOI 100 for 6 h and analyzed by Western blot for pAblT735 and c-Abl. β-actin served as loading control. (B) Untreated AGS cells and AGS cells expressing TAP-Ablwt or TAP-AblTA were either left untreated (mock) or infected with Hp at a MOI 100. The scattering phenotype was documented using phase contrast microscopy. (C) Untreated AGS cells and AGS cells stably transfected with control shRNA (shCtrl) or c-Abl shRNA (shAbl) were lysed and analyzed by Western blotting for c-Abl and GAPDH expression (D) AGS cells stably transfected with control shRNA (shCtrl) or c-Abl shRNA (shAbl) were either left untreated (mock) or infected with Hp at a MOI 100 for 6 h. Scattering phenotype was documented using phase contrast microscopy. (E) AGS cells stably transfected with control (shCtr) or Abl shRNA (shAbl) were left untreated (−) or infected with Hp wt for 48 h. Percent apoptosis was calculated by analyzing annexin single-positive and annexin/7AAD positive cells. (DOCX 276 kb) [file 12964_2019_323_MOESM5_ESM.docx]

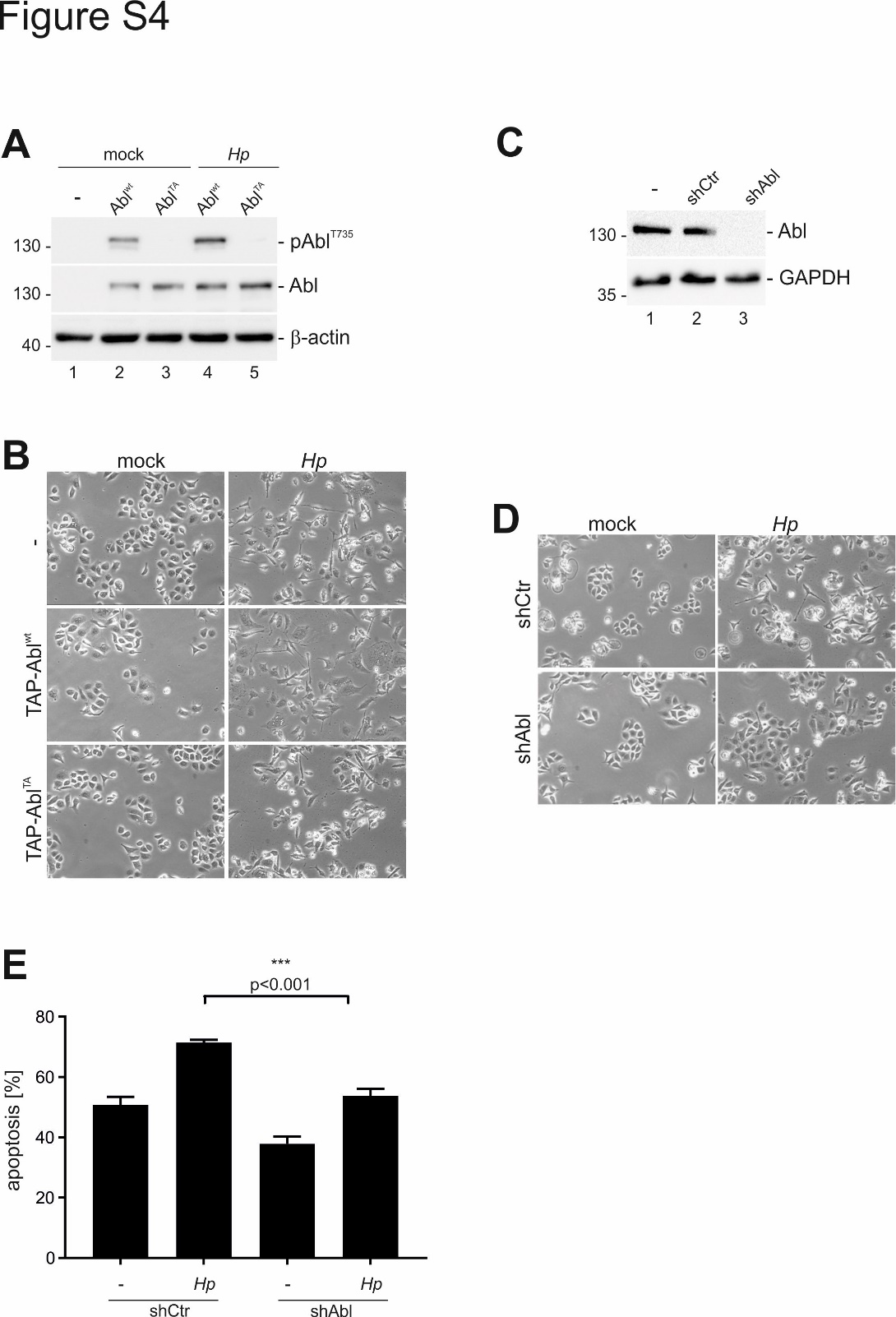


**Figure S4: Generation of stable AGS cell lines. (A)** Untreated AGS cells and AGS cells transfected with TAP-Abl^wt^ or TAP-Abl^TA^ were either left untreated (mock) or infected with *Hp* at a MOI 100 for 6 hours and analyzed by Western blot for pAbl^T735^ and c-Abl. β-actin served as loading control. **(B)** Untreated AGS cells and AGS cells expressing TAP-Abl^wt^ or TAP-Abl^TA^ were either left untreated (mock) or infected with *Hp* at a MOI 100. The scattering phenotype was documented using phase contrast microscopy. **(C)** Untreated AGS cells and AGS cells stably transfected with control shRNA (shCtrl) or c-Abl shRNA (shAbl) were lysed and analyzed by Western blotting for c-Abl and GAPDH expression **(D)** AGS cells stably transfected with control shRNA (shCtrl) or c-Abl shRNA (shAbl) were either left untreated (mock) or infected with *Hp* at a MOI 100 for 6 hours. Scattering phenotype was documented using phase contrast microscopy. **(E)** AGS cells stably transfected with control (shCtr) or Abl shRNA (shAbl) were left untreated (-) or infected with *Hp* wt for 48 hours. Percent apoptosis was calculated by analyzing annexin single-positive and annexin/7AAD positive cells.
