# Supplementary material for: Helicobacter pylori-controlled c-Abl localization promotes cell migration and limits apoptosis
Source: Cell Commun Signal. 2019 Jan 31;17:10. doi: 10.1186/s12964-019-0323-9 (PMC6357398; doi:10.1186/s12964-019-0323-9)
Supplement: Supplementary file 6 — Figure S5. Gleevec decreases Hp pathology. C57BL/6 mice were infected with Hp PMSS1 for two months, were supplied with STI-571 or remained untreated (control). Representative sections of the gastric tissues are shown. (DOCX 261 kb) [file 12964_2019_323_MOESM6_ESM.docx]

**
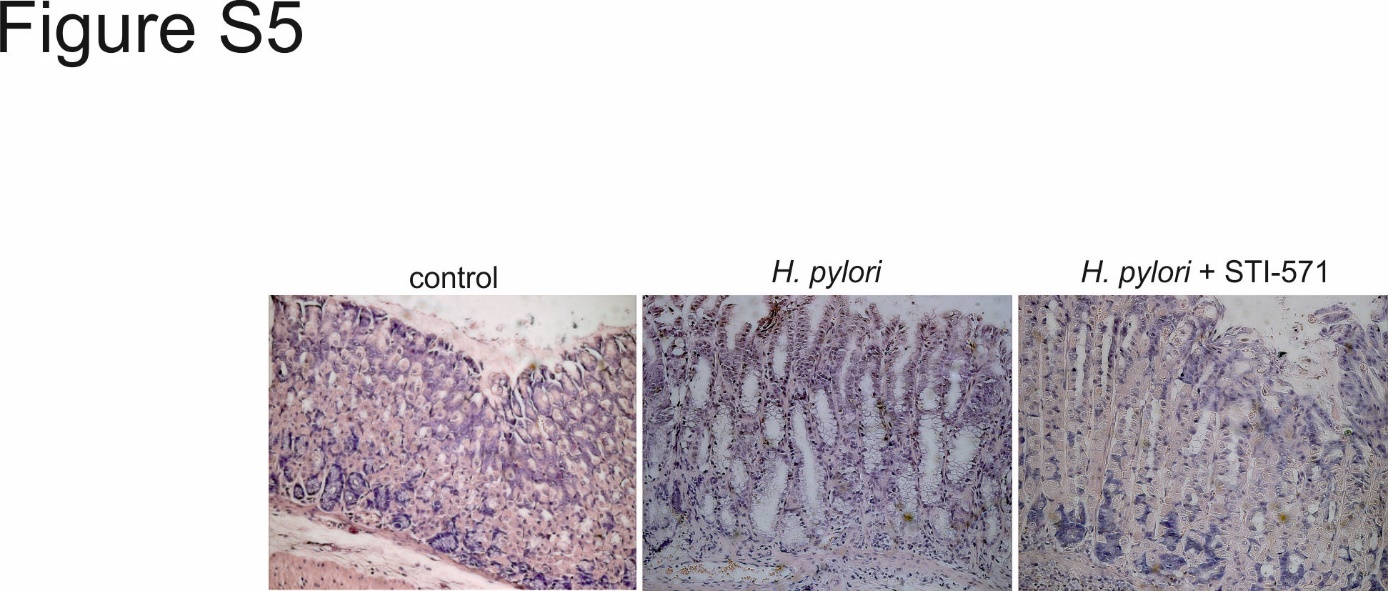
**

**Figure S5: Gleevec decreases *Hp* pathology.** C57BL/6 mice were infected with *Hp* PMSS1 for two months, were supplied with STI-571 or remained untreated (control). Representative sections of the gastric tissues are shown.
